# Novel Small Molecule Growth Inhibitor Affecting Bacterial Outer Membrane Reduces Extraintestinal Pathogenic Escherichia coli (ExPEC) Infection in Avian Model

**DOI:** 10.1128/Spectrum.00006-21

**Published:** 2021-09-01

**Authors:** Dipak Kathayat, Yosra A. Helmy, Loic Deblais, Vishal Srivastava, Gary Closs, Rahul Khupse, Gireesh Rajashekara

**Affiliations:** a Center for Food Animal Health, Department of Animal Sciences, The Ohio State University, Wooster, Ohio, USA; b College of Pharmacy, University of Findlay, Findlay, Ohio, USA; Universidade de Sao Paulo

**Keywords:** APEC, ExPEC, small molecule, Lpt system, LPS, *Lactobacillus*, oleate, lipopolysaccharide, LptD/E

## Abstract

Avian pathogenic Escherichia coli (APEC), a subgroup of extraintestinal pathogenic E. coli (ExPEC), causes colibacillosis in chickens and is reportedly implicated in urinary tract infections and meningitis in humans. A major limitation for the current ExPEC antibiotic therapy is the development of resistance, and antibacterial drugs that can circumvent this problem are critically needed. Here, we evaluated eight novel membrane-affecting anti-APEC small molecule growth inhibitors (GIs), identified in our previous study, against APEC infection in chickens. Among the GIs tested, GI-7 (the most effective), when administered orally (1 mg/kg of body weight), reduced the mortality (41.7%), severity of lesions (62.9%), and APEC load (2.6 log) in chickens. Furthermore, GI-7 administration at an optimized dose (60 mg/liter) in drinking water also reduced the mortality (14.7%), severity of lesions (29.5%), and APEC load (2.2 log) in chickens. The abundances of *Lactobacillus* and oleate were increased in the cecum and serum, respectively, of GI-7-treated chickens. Pharmacokinetic analysis revealed that GI-7 was readily absorbed with minimal accumulation in the tissues. Earlier, we showed that GI-7 induced membrane blebbing and increased membrane permeability in APEC, suggesting an effect on the APEC membrane. Consistent with this finding, the expression of genes essential for maintaining outer membrane (OM) integrity was downregulated in GI-7-treated APEC. Furthermore, decreased levels of lipopolysaccharide (LPS) transport (Lpt) proteins and LPS were observed in GI-7-treated APEC. However, the mechanism of action of GI-7 currently remains unknown and needs further investigation. Our studies suggest that GI-7 represents a promising novel lead compound that can be developed to treat APEC infection in chickens and related human ExPEC infections.

**IMPORTANCE** APEC is a subgroup of ExPEC, and genetic similarities of APEC with human ExPECs, including uropathogenic E. coli (UPEC) and neonatal meningitis E. coli (NMEC), have been reported. Our study identified a novel small molecule growth inhibitor, GI-7, effective in reducing APEC infection in chickens with an efficacy similar to that of the currently used antibiotic sulfadimethoxine, notably with an 8-times-lower dose. GI-7 affects the OM integrity and decreases the Lpt protein and LPS levels in APEC, an antibacterial mechanism that can overcome the antibiotic resistance problem. Overall, GI-7 represents a promising lead molecule/scaffold for the development of novel antibacterial therapies that could have profound implications for treating APEC infections in chickens, as well as human infections caused by ExPECs and other related Gram-negative bacteria. Further elucidation of the mechanism of action of GI-7 and identification of its target(s) in APEC will benefit future novel antibacterial development efforts.

## INTRODUCTION

Avian pathogenic Escherichia coli (APEC), a subgroup of extraintestinal pathogenic E. coli (ExPEC), is a foodborne zoonotic pathogen and a source of extraintestinal infections in humans (1–4). In particular, APEC shares a genetic similarity with human ExPECs (uropathogenic E. coli [UPEC] and neonatal meningitis E. coli [NMEC]), possesses UPEC- and NMEC-associated virulence genes, and is able to cause urinary tract infections and meningitis in rodent models (1). Furthermore, the detection of APEC-specific colicin V (ColV) plasmids in human ExPEC isolates suggests a possible zoonotic transmission of APEC from poultry to humans (4). Moreover, poultry hatcheries and poultry products are regarded as reservoirs for antibiotic-resistant E. coli strains (5). Therefore, the consumption of contaminated poultry products could contribute to the transfer of antibiotic-resistant E. coli strains, as well as antibiotic resistance genes (ARGs), to human pathogens, which can make human infections difficult to treat (1, 5). Thus, APEC is a pathogen of significant importance to human health.

APEC is a very common bacterial pathogen of poultry, including chickens, turkeys, and ducks, and of many other avian species (6, 7). It is widely prevalent in all age groups of chickens (9.52 to 36.73%), with specifically high prevalence in adult layer birds (36.73%) (8). It is estimated that at least 30% of the commercial flocks in the United States at any point in time are affected by APEC (9). APEC causes a wide range of localized and systemic infections in poultry, including yolk sac infection, omphalitis, respiratory tract infection, swollen head syndrome, septicemia, polyserositis, coligranuloma, enteritis, cellulitis, and salphingitis, commonly referred to as avian colibacillosis (10). Colibacillosis can result in high morbidity and mortality (up to 20%), decreased meat and egg production, and increased condemnation (up to 43%) of carcasses at slaughter (1, 11, 12), thus leading to extensive economic losses in all facets of the global poultry industry (13).

At present, antibiotics (tetracyclines, sulfonamides, and aminoglycosides) are commonly used to control colibacillosis in poultry (14). However, evidence exists that APEC strains are becoming more resistant to these antibiotics, indicating that the control of colibacillosis is likely to become even more challenging in the future (9). Up to 92% of APEC isolates characterized in the United States, Europe, and Australia were resistant to three or more antibiotics, particularly against tetracyclines, aminoglycosides, and sulfonamides (13). Recently, APEC isolates with the *mcr-1* gene, which confers resistance to colistin (one of the drugs of last resort to treat human infectious diseases), have been detected (15). Furthermore, APEC isolates with extended-spectrum-β-lactamase (ESBL) genotypes with resistance to β-lactam antibiotics have been also detected (16, 17). Moreover, the current vaccine (Poulvac E. coli) designed to prevent avian colibacillosis is only effective against homologous APEC serotype O78 challenge and fails to confer protection against heterologous serotypes (O1, O2, O8, O15, O35, O109, and O115) commonly associated with colibacillosis (13). Therefore, there is an urgent need for the development of new anti-APEC therapeutics for the effective control of this key endemic avian disease, including control of emerging antibiotic-resistant APEC infections.

Our previous study identified 11 small molecule growth inhibitors (GIs) with bactericidal activity against APEC (18). Previously, these GIs were designated SM1 to SM11 (18); however, in the current study they are referred as GI-1 to GI-11. Previously, using microscopy and *in vitro* studies (crystal violet uptake and loss of 260-/280-nm-absorbing materials) we showed that these GIs induced membrane defects like membrane blebbing, disruption of membrane, and increased membrane permeability (18), suggesting an effect on the APEC membrane. Antibacterial compounds that target the outer membrane (OM) of Gram-negative bacteria can counter the resistance problem (19, 20). The OM acts as a shield for bacteria by providing a permeability barrier, thus precluding the entry of antibiotics in bacteria (19, 21). The OM in Gram-negative bacteria is critical for viability and virulence and plays a key role in resistance to antibiotics (19, 21). The OM is composed of lipopolysaccharides (LPS), β-barrel proteins, phospholipids, and lipoproteins (21). These components are transported from the inner membrane (IM) and assembled at the OM by different assembly/transport complexes, including LPS transport (Lpt), β-barrel assembly (Bam), maintenance of lipid asymmetry (Mla), and localization of lipoproteins (Lol) complexes (21). Inhibition of the function of these transport/assembly complexes leads to a defective OM; therefore, the OM is an attractive target for the development of effective antibacterial drugs to circumvent the resistance problem (21). Different OM-targeting antibacterial compounds have been identified with activity against multidrug-resistant (MDR) Gram-negative pathogens, particularly E. coli and Pseudomonas aeruginosa (19, 21). The β-hairpin macrocyclic peptides (JB-95, murepavadin, and L27-11) and thanatin (insect-derived antimicrobial peptide) target the Lpt pathway, specifically LptD, thus inhibiting the transport of LPS to the OM (22–25). Similarly, darobactin (antibiotic derived from *Photorhabdus*), MRL-494 (synthetic compound), and LlpA (lectin-like bacteriocin) target the Bam complex, particularly BamA, resulting in inhibition of the assembly of OM proteins (26–28). Arenicin-3, a peptide derived from Arenicola marina, inhibits the Mla pathway, specifically MlaC, leading to perturbation of the OM phospholipid equilibrium (29). Notably, resistance is less likely to occur against OM-targeting antibacterial compounds, as they avoid the most common mechanisms for resistance development in bacteria, which are the entry barriers that prevent access by antibiotics and the efflux pumps that eliminate antibiotics from bacterial cells (19, 21). Moreover, OM-acting antibacterial compounds can make resistant bacteria susceptible to antibiotics when used in combination with antibiotics (19, 20, 30). Therefore, antibacterial compounds targeting the OM are promising leads for the development of novel therapeutics to circumvent the resistance problem in ExPECs and other Gram-negative bacteria.

In this study, we evaluated the efficacy and safety of eight membrane-affecting anti-APEC small molecule GIs identified in our earlier study (18). We extensively evaluated the most effective GI, GI-7, for its effect on the cecal microbiome and serum metabolome of chickens (31, 32). We optimized the delivery of GI-7 in drinking water of chickens and assessed the efficacy and safety under conditions mimicking the field settings. Furthermore, we explored the potential mechanism of action (MOA) of GI-7 by using bacterial cytological profiling (BCP) (18), gene expression, and immunoblot studies followed by *in silico* docking studies (22, 33, 34). Our findings demonstrate that GI-7 affects the OM integrity and decreases the Lpt protein and LPS levels in APEC through a mechanism that remains currently unknown and therefore warrants further investigation. Overall, GI-7 represents a promising antibacterial compound and scaffold for the development of novel antibacterial therapies for effectively treating APEC and other ExPEC infections and circumventing the antibiotic resistance problem.

## RESULTS

### Three GIs reduced the mortality, lesion severity, and APEC load in chickens.

**FIG 1:**
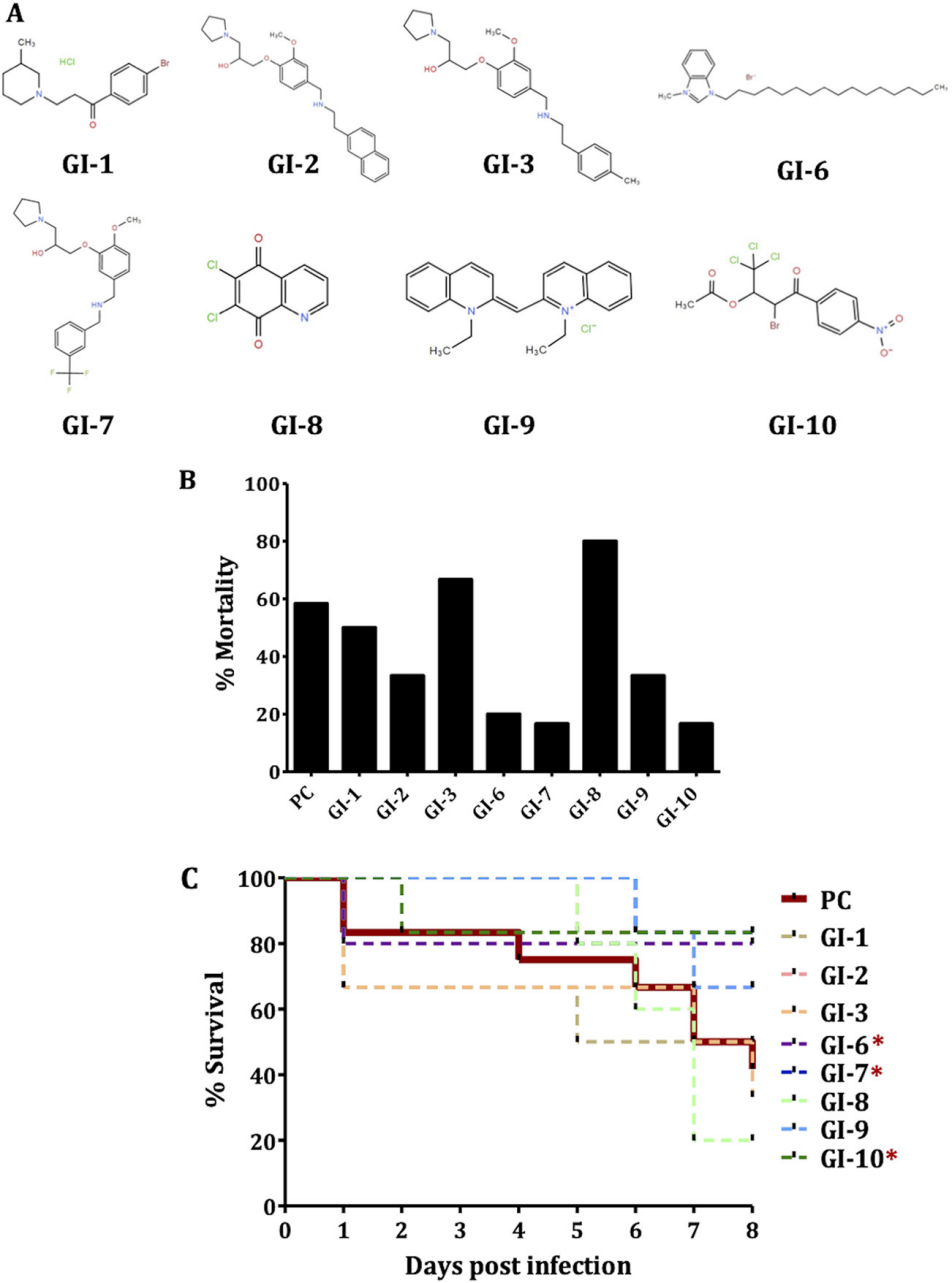
(A) Structures of growth inhibitors (GIs) tested against APEC infection in chickens in this study. (B) Mortality (%) in GI-treated groups compared to PC (infected and vehicle-treated; positive control) group. (C) Survival curve of chickens in PC- and GI-treated groups. *, *P* ≤ 0.1; log rank test.

We tested eight growth inhibitors (GIs) belonging to five different chemical scaffolds (pyrrolidinyl, GI-2, GI-3 and GI-7; imidazole, GI-6; quinoline, GI-8 and GI-9; piperidine, GI-1; and nitrophenyl, GI-10) (Fig. 1A) that were identified in our previous study (18) against APEC infection in chickens. In our earlier study, by screening 4,182 compounds, a total of 40 compounds were identified as having an inhibitory effect on the growth of APEC serotype O78 at a 100 μM concentration. The eight GIs selected in this study were effective against APEC *in vitro*, in cultured epithelial/macrophage cells, and in wax moth larva. These compounds were minimally toxic or nontoxic to eukaryotic cells and wax moth larva (18). The MICs of these GIs against APEC O78 ranged from 12.5 μM to 200 μM (GI-6 and GI-8, 12.5 μM; GI-7, GI-9, and GI-10, 25 μM; GI-2 and GI-3, 100 μM; and GI-1, 200 μM) (18). To measure the mortality reduction in chickens, the mortality rates observed in GI-treated groups were subtracted from the mortality rate in the positive-control (PC; infected and vehicle-treated) group (58.3%). GI-7 and GI-10 reduced the mortality by 41.7% and GI-6 reduced the mortality by 38.3% compared to the PC group (Fig. 1B). GI-2, GI-9, and GI-1 were less effective and reduced the mortality by 25%, 25%, and 8.3%, respectively. Consistent with the reduction of mortality, GI-7, GI-10, and GI-6 also prolonged the survival of chickens (*P* ≤ 0.1) (Fig. 1C).

**FIG 2:**
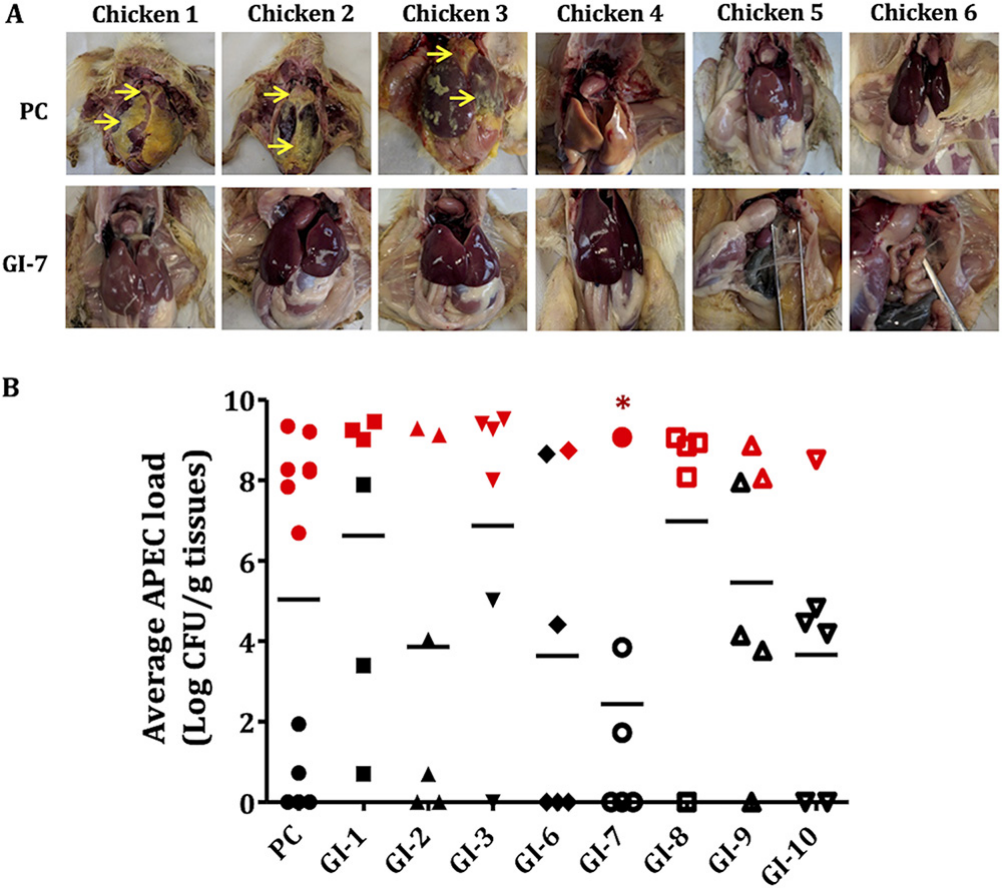
(A) APEC lesions in PC (infected and vehicle-treated; positive control) and GI-7-treated groups. Images of the internal organs of all the chickens from the PC and GI-7 groups are included. In the PC group, chickens 1, 2, and 3 showed severe APEC lesions (yellow arrows), while only chicken 1 in the GI-7 groups showed mild APEC lesions. (B) Mean APEC loads in PC and GI-treated chickens. APEC loads were quantified in internal organs (liver, heart, lung, and kidney) of chickens and are displayed as the mean of all organs in each chicken. Red symbols show APEC loads in chickens that died within 7 days postinfection (dpi), black symbols show APEC loads in live chickens sacrificed and necropsied at 7 dpi, and horizontal bars show the mean value for each group. *, *P* < 0.05; Mann-Whitney U test.

The severity of the APEC lesions in liver, heart, lung, and air sacs of chickens was assessed, and the mean lesion score of each organ was calculated for each group. The cumulative mean lesion score was calculated by adding the mean lesion score of each organ. The PC (infected and vehicle-treated) chickens had a cumulative mean lesion score of 6.2, and only GI-7-treated chickens had a significantly lower (*P* < 0.05) cumulative mean lesion score of 2.3, while GI-2- and GI-6-treated chickens had cumulative mean lesion scores of 5.3 (*P* > 0.05) (Table 1). The treatment of APEC-infected chickens with GI-2, GI-6, and GI-7 reduced the lesion severity in liver, heart, air sacs, and lung by 14.5% to 62.9% (on average) in infected chickens (Table 1). The highest reduction in lesion severity was observed in the GI-7-treated group (52.9% to 83.3%), followed by the GI-6-treated (20% to 40%) and GI-2-treated (12% to 33.3%) groups. GI-7 treatment significantly (*P* < 0.05) reduced the severity of liver lesions by 60%, heart lesions by 60%, lung lesions by 83.3%, and air sac lesions by 52.9% (Table 1). Images showing the APEC lesions in internal organs of GI-7-treated (most effective GI) and PC chickens are displayed in Fig. 2A.

**TABLE 1:** APEC lesion scores and reduction of lesion severity in GI-treated chickens

Trial, treatment^*a*^	Mean lesion score (% reduction in severity) in^*b*^:				Cumulative lesion score
	Liver	Heart	Lung	Air sacs	
Efficacy					
GI-1	2.0 (−20)	2.0 (4)	0.7 (33.3)	1.5 (−5.9)	6.2
GI-2	1.7 (0)	1.8 (12)	0.7 (33.3)	1.2 (17.7)	5.3
GI-3	2 (−20)	2 (4)	1 (0)	1.8 (−29.4)	6.8
GI-6	1 (40)	1.7 (20)	0.7 (33.3)	2 (−41.2)	5.3
GI-7	0.7 (60)*	0.8 (60)*	0.2 (83.3)*	0.7 (52.9)*	2.3
GI-8	2.8 (−68)	3.8 (−82.4)	2 (−100)	3 (−111.8)	11.6
GI-9	2.5 (−50)	3.3 (−60)	2 (−16.7)	2.7 (−88.2)	9.7
GI-10	1.3 (20)	2.2 (−4)	1 (0)	1.7 (−17.6)	6.2
PC	1.7	2.1	1	1.4	6.2

GI-7 dose-response					
20 mg/liter	2 (0)	2.7 (−9.5)	1.8 (10)	2.3 (1.4)	8.8
40 mg/liter	2 (0)	2.6 (−5.4)	1.4 (30)	1.4 (27.1)	7.7
60 mg/liter	1.7 (13.6)	2.1 (15.2)	1.4 (36.3)*	1.7 (33.8)*	6.6
PC	2	2.5	2	2.3	8.8

Comparative efficacy					
GI-7	2.3 (7.2)	3.2 (10.2)*	1.3 (29.5)***	3.0 (9.9)	9.8
SDM	2.0 (19.2)*	2.8 (20.8)**	1.2 (36.9)***	2.5 (25.7)**	8.5
PC	2.5	3.6	1.8	3.7	11.3

a PC, positive control (infected and vehicle-treated chickens); SDM, sulfadimethoxine.

b Statistical significance compared to PC chickens is shown by asterisks: *, *P* < 0.05; **, *P* < 0.01; ***, *P* < 0.001.

The APEC burden was quantified in different internal organs (liver, heart, lung, and kidney) of the chickens. Treatment with GI-2, GI-6, GI-7, and GI-10 reduced the APEC loads in internal organs by 1.2 to 2.6 log/g of tissue (average APEC load across all internal organs combined) compared to the APEC load in the PC (infected and vehicle-treated) group (Fig. 2B). The average APEC load was determined by calculating the mean APEC load for all internal organs of each chicken. GI-7 most effectively reduced the APEC loads in internal organs (2.3 to 3.2 log) (*P* < 0.05), followed by GI-10 (0.8 to 2.4 log), GI-6 (1.2 to 1.9 log), and GI-2 (0.8 to 1.7 log) (Fig. 2B and Table S4 in the supplemental material). GI-7 treatment significantly (*P* < 0.05) reduced the APEC load in the liver by 2.5 log, in the lung by 2.4 log, in the heart by 2.3 log, and in the kidney by 3.2 log (Table S4). None of the GIs affected the body weight gain (BWG) of chickens (*P* > 0.05) compared to the BWG in the NC (noninfected and nontreated) group, except GI-2 (Table S5). GI-7-treated chickens had BWG similar to that of NC chickens.

### Resistance to GIs was not observed in APEC isolated from treated chickens.

Randomly selected APEC colonies (*n* = 24/group) isolated from internal organs of chickens treated with GI-6, GI-7, and GI-10 were tested for their resistance against GIs. The same MICs and minimal bactericidal concentrations (MBCs) were observed before and after the treatment for all three GIs that showed impacts against APEC infection in chickens as described above (MICs/MBCs were as follows: GI-6, 12.5/25 μM; GI-7, 25/50 μM; and GI-10, 25/50 μM) (18). This is consistent with our earlier *in vitro* results where no resistance to GI-7 was detected when a high inoculum of APEC (10^9^ CFU) was incubated with twice the lethal concentration (2× MBC) of GI-7 or passaged 15 times with a sublethal concentration (0.75× MIC) of GI-7 (18).

### *Lactobacillus* abundance was significantly increased in GI-7-treated chickens.

**FIG 3:**
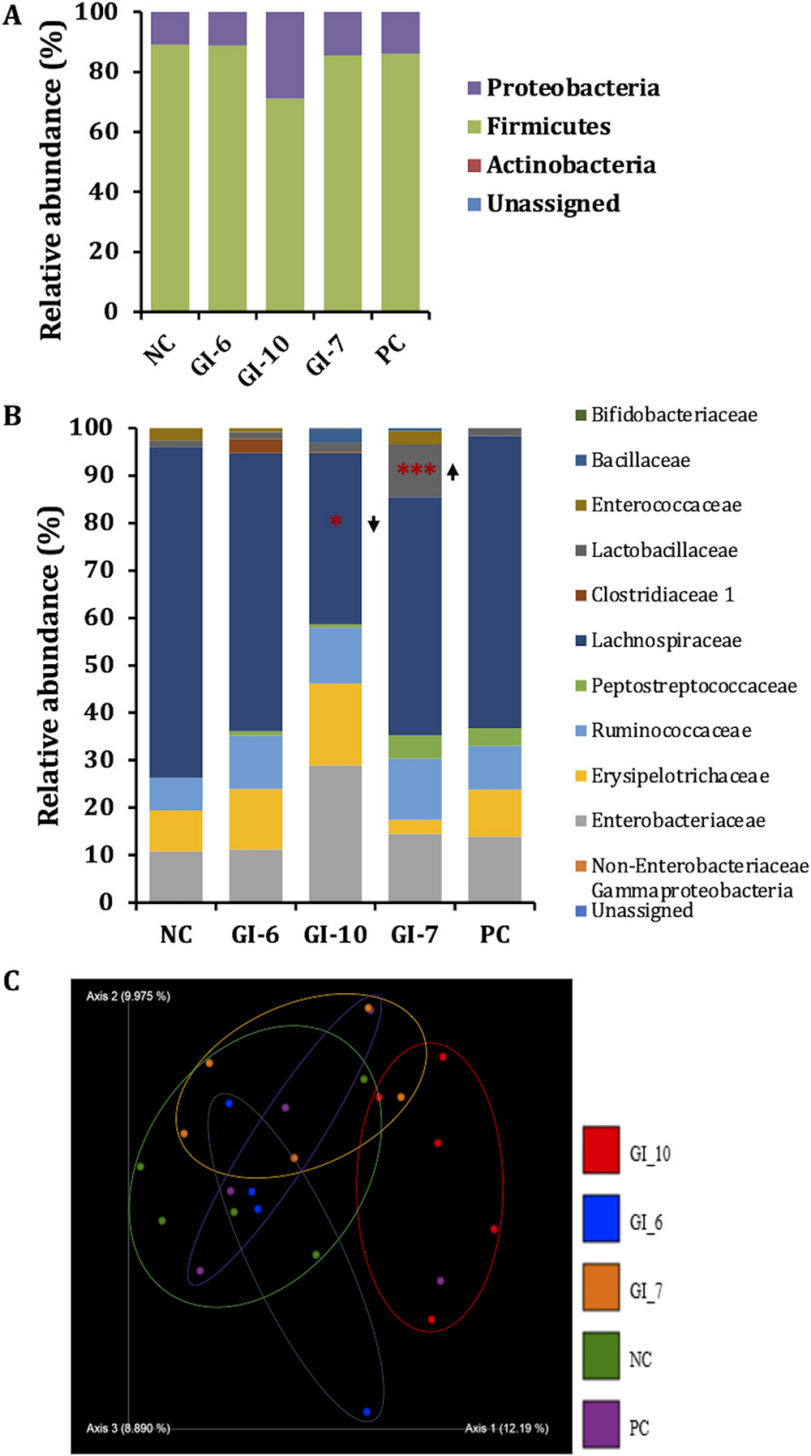
(A) Microbial relative abundances at the phylum level in growth inhibitor (GI)-treated, NC (noninfected and nontreated; negative control), and PC (infected and vehicle-treated; positive control) groups. (B) Microbial relative abundances at the family level in GI-treated, NC, and PC groups. *, *P* < 0.05; ***, *P* < 0.001; Mann-Whitney U test. (C) Bray-Curtis dissimilarity graph comparing the microbial similarity/dissimilarity between GI-treated, PC, and NC groups.

We investigated the effects of GIs on gut microbiota through metagenomic analysis of the cecal microbiota of chickens (31). GI-treated chickens displayed a cecum microbial composition that was similar to that of the NC (noninfected and nontreated) group at the phylum level, except for the GI-10-treated group (Fig. 3A). The *Firmicutes* abundance was reduced (89% to 71%) and the *Proteobacteria* abundance was increased (11% to 29%) in GI-10-treated chickens. At the class level, the *Bacilli* abundance was increased (4% to 14%) in GI-7-treated chickens, while the *Clostridia* abundance was decreased (77% to 49%) in GI-10-treated chickens. Similar trends of increased *Lactobacillales* (4% to 14%) in GI-7-treated chickens and decreased *Clostridiales* (77% to 49%) in GI-10-treated chickens were observed at the order level. At the family level, the *Lactobacillaceae* population was significantly increased (1% to 11%) in GI-7-treated chickens, while the *Lachnospiraceae* population was significantly decreased (68% to 36%) in GI-10-treated chickens (Fig. 3B and Table S6). Specifically, *Lactobacillus* species (Lactobacillus zeae, 0% to 7%, and Lactobacillus helveticus, 0% to 4%) abundances were increased in GI-7-treated chickens and *Lachnoclostridium* (29.7% to 8.1%) abundance was decreased in GI-10-treated chickens (Table 2). Bacterial genera *Blautia*, *Shuttleworthia*, and *Butyricicoccus* were uniquely detected in GI-7-treated chickens, whereas *Bifidobacterium* (species *longum*), CAG-56, *Clostridioides*, *Negativibacillus*, and UBA1819 were uniquely detected in GI-10-treated chickens (Table 2). *Ruminococcaceae* member UCG-005 was uniquely detected in GI-6-treated chickens (Table 2). Interestingly, the abundance of *Flavonifractor* was increased in all GI-treated groups compared to its abundance in the PC (infected and vehicle-treated) group (Table 2). The microbial richness was significantly (*P* < 0.05) higher in GI-10-treated chickens than in GI-7-treated, PC, and NC groups (Fig. S4A). The microbial communities in GI-10-treated chickens were dissimilar (clustered differently) to those in NC, PC, GI-6-treated, and GI-7-treated chickens (Fig. 3C).

**TABLE 2:** Microbial composition in different treatment groups at genus level

Microbe	Abundance (%) in cecal microbiota of indicated treatment group				
	NC	GI-6	GI-10	GI-7	PC
Escherichia *-Shigella*	8.9	6.5	26.6	12.3	8.5
[*Clostridium*] *inoculum* group	1.3	0.0	2.8	0.8	0.0
*Erysipelatoclostridium*	0.0	0.0	1.1	0.0	0.0
[*Eubacterium*] *coprostanoligenes* group	1.5	0.0	0.0	1.5	0.0
UBA1819	0.0	0.0	1.1	0.0	0.0
*Ruminococcaceae* UCG-014	0.0	1.2	0.4	0.0	0.0
*Ruminococcaceae* UCG-005	0.0	2.7	0.0	0.0	0.0
*Ruminiclostridium* 9	0.0	0.0	0.0	0.0	0.6
*Oscillibacter*	0.5	1.7	0.8	3.0	5.1
*Negativibacillus*	0.0	0.0	1.6	0.0	0.0
*Flavonifractor*	3.0	5.0	7.7	3.8	0.0
*Caproiciproducens*	0.4	0.7	0.3	0.0	1.5
*Butyricicoccus*	0.0	0.0	0.0	4.0	2.1
*Anaerotruncus*	1.6	0.0	0.0	0.0	0.0
*Clostridioides*	0.0	0.0	0.7	0.0	0.0
[*Ruminococcus*] *torque*s group	9.0	27.3	13.9	24.8	15.2
[*Ruminococcus*] *gauvreauii* group	1.3	0.0	0.03	3.0	0.00
*Tyzzerella*	2.1	0.5	0.4	0.0	0.7
*Shuttleworthia*	0.0	0.0	0.0	1.7	0.0
*Sellimonas*	0.0	1.0	0.0	0.0	1.6
*Lachnoclostridium*	29.7	15.9	8.1	13.0	18.5
CAG-56	0.0	0.0	0.7	0.0	0.0
*Blautia*	0.0	0.0	0.0	1.8	0.0
*Anaerostipes*	0.4	0.0	1.4	0.0	2.0
*Clostridium sensu stricto* 1	0.0	2.9	0.3	0.0	0.0
*Lactobacillus*	1.4	1.5	1.9	11.02^*a*^^,^^*b*^	1.6
*Enterococcus*	2.6	0.8	0.0	2.9	0.0
*Bacillus*	0.0	0.0	3.0	0.6	0.0
*Bifidobacterium*	0.0	0.0	0.1	0.0	0.0

a Significantly (*P* < 0.05) altered in GI-treated group compared to NC (noninfected and nontreated; negative control) group.

b Significantly (*P* < 0.05) altered in GI-treated group compared to PC (infected and vehicle-treated; positive control) group.

No significant alterations in the microbial composition were observed between the PC and NC groups, except for an increased abundance of P*eptostreptococcaceae* (0% to 3.7%) and decreased abundance of *Enterococcaceae* (2.6% to 0%) in PC chickens (Fig. 3B). Specifically, *Oscillibacter* (0.5% to 5.1%), *Sellimonas* (0% to 1.6%), and *Anaerostipes* (species *butyraticus*; 0.4% to 2%) were increased while *Anaerotruncus* (1.6% to 0%), *Flavonifractor* (3% to 0%), *Enterococcus* (species *cecorum*; 2.6% to 0%), and *Tyzzerella* (2.1% to 0.7%) were decreased in APEC-infected chickens (Table 2).

### Oleate was elevated in GI-7-treated chickens and l-arginine was elevated in APEC-infected chickens.

The metabolomic profiles of the serum of chickens treated with GIs were determined to assess the effects of GIs on the serum metabolites of chickens (32). The PC (infected and vehicle-treated) chickens had elevated levels (1.5- to 5.3-fold) of l-arginine, IMP, acetoacetate, (5)-methylmalonate-semialdehyde, and 2-oxobutanoate compared to the levels in NC (noninfected and nontreated) chickens (Table 3). Only a few metabolites were altered in GI-7-treated chickens. The level of oleate was elevated (2-fold; *P* < 0.05) in GI-7-treated chickens, while the level of l-saccharopine was decreased (greater than −2.7-fold; *P* < 0.05) compared to the levels in NC and PC chickens (Table 3). The levels of sucrose, α,α-trehalose, geranyl diphosphate, β-nicotinate d-ribonucleotide, l-saccharopine, 4-(-3-pyridyl)-butanoate, urate, l-lysine, 5-amino-1-(5-phospho-d-ribosyl)imidazole-4-carboxamide, coproporphyrinogen III, inosine, and thyronamine were elevated (1.9- to 5.4-fold; *P* < 0.05) in GI-10-treated chickens compared to the levels in NC chickens (Table 3). Compared to PC chickens, the levels of 4*S*/7*S*-hydroperoxy 17*R*-docosahexaeonate, aspirin-triggered resolvins D1/D2/D3/D4, thymine, 2′-deoxyinosine, 4-imidazolacetate, and uracil were elevated (2.1- to 3.8-fold; *P* < 0.05) in GI-10-treated chickens (Table 3). Interestingly, the levels of xanthine and xanthosine were elevated (3.7 to 5.6-fold; *P* < 0.05) in GI-10-treated chickens compared to the levels in both NC and PC chickens (Table 3). Similarly, nicotine-glucuronide, 4-hydroxy-4(3-pyridyl)-butanoate, 3-methoxy-4-hydroxyphenylglycoaldehyde, homovanillate, and d-sorbitol levels were elevated (1.4 to 2.7-fold; *P* < 0.05) in GI-6-treated chickens compared to the levels in NC and PC chickens (Table 3). Overall, only the gamma-linolenate biosynthesis pathway was affected by GI-7 treatment, while the urate biosynthesis/inosine 5′-phosphate degradation, adenosine nucleotide degradation, purine ribonucleoside degradation to ribose-1-phosphate, nicotine degradation IV/III, tRNA charging, lysine degradation I (saccharopine), and guanosine nucleotide degradation pathways were affected by GI-10 and GI-6 treatments. Principal-component analysis (PCA) revealed no distinct clustering of metabolome profiles between the treatment groups (Fig. 3C and Fig. S4B).

**TABLE 3:** Metabolites that were significantly altered in APEC-infected and growth inhibitor-treated chickens

Metabolite^*a*^	Fold change in indicated treatment group^*b*^			
	GI-6	GI-10	GI-7	PC
**Xanthine**	2.8	3.7	NA	−1.5
**Xanthosine**	3.6	5.6	NA	1.0
3-Mercaptopyruvate	2.1	1.9	NA	2.0
Sucrose	2.4	2.6	NA	1.7
Linoleate	NA	NA	−1.8	−2.2
α,α-Trehalose	2.4	2.6	NA	1.7
Nicotine-glucuronide	2.7	1.5	NA	1.6
**4-Hydroxy-4-(3-pyridyl)-butanoate**	1.9	1.3	NA	1.4
**3-Methoxy-4-hydroxyphenylglycoaldehyde**	1.9	1.3	NA	1.4
19-Hydroxytestosterone	NA	NA	−2.4	−3.2
Homovanillate	1.9	1.3	NA	1.4
Geranyl diphosphate	3.7	5.4	NA	3.6
β-Nicotinate d-ribonucleotide	3.7	5.4	NA	3.6
l-Saccharopine	2.2	3.0	NA	1.7
4-(3-Pyridyl)-butanoate	1.8	1.9	NA	1.4
**Oleate**	NA	NA	2.0	1.4
**Urate**	2.5	3.0	NA	2.1
**l-Lysine**	1.8	2.2	NA	1.4
5-Amino-1-(5-phospho-d-ribosyl)imidiazole-4-carboxamide	2.6	3.3	NA	2.1
d-Sorbitol	2.0	1.3	NA	1.4
15*S*-Hydroxypentaenoate	NA	NA	−1.3	−1.4
**l-Arginine**	2.0	2.7	1.0	3.2
4*S*-Hydroperoxy,17*R*-*H*-docosahexaenoate	−1.1	1.2	NA	−1.7
7*S*-Hydroperoxy,17*R*-*H*-docosahexaenoate	−1.1	1.2	NA	−1.7
Aspirin-triggered resolvin D1	−1.1	1.2	NA	−1.7
(15*R*)-Hydroxypentaenoate	NA	NA	−1.3	−1.4
Aspirin-triggered resolvin D2	−1.1	1.2	NA	−1.7
Aspirin-triggered resolvin D3	−1.1	1.2	NA	−1.7
Aspirin triggered resolvin D4	−1.1	1.2	NA	−1.7
Coproporphyrinogen III	2.5	2.9	NA	2.0
**Inosine**	2.1	2.4	NA	1.5
α-Carboxyethylhydroxychroman	NA	NA	−2.8	1.0
Thyronamine	2.1	2.4	NA	1.5
Thymine	1.1	1.3	NA	−2.8
2′-Deoxyinosine	1.1	1.3	NA	−2.8
4-Imidazolacetate	1.1	1.3	NA	−2.8
Cytidine	1.8	2.6	NA	−1.1
l-Saccharopine	NA	NA	−2.8	1.0
IMP	1.8	1.6	NA	2.0
Uracil	1.2	1.6	NA	−1.6
Acetoacetate	3.2	2.9	NA	5.3
(*S*)-Methylmalonate-semialdehyde	3.2	2.9	NA	5.3
2-Oxo-butanote	3.2	2.9	NA	5.3

a Boldface indicates the metabolites that were significantly (*P* < 0.05) altered as analyzed using Progenesis QI.

b Fold change was calculated in comparison to the abundance of the metabolite in serum of NC (noninfected and nontreated; negative control) chickens. Fold increase (for metabolite with abundance higher than NC group) = abundance of metabolite in GI or PC (infected and vehicle-treated chickens; positive control) group/abundance of metabolite in NC group. Fold decrease (for metabolite with abundance lower than NC group) = abundance of metabolite in NC group/abundance of metabolite in GI or PC group. NA, not available.

### GI-7 administered at 60 mg/liter in drinking water reduced mortality, lesion severity, and APEC load in chickens.

**FIG 4:**
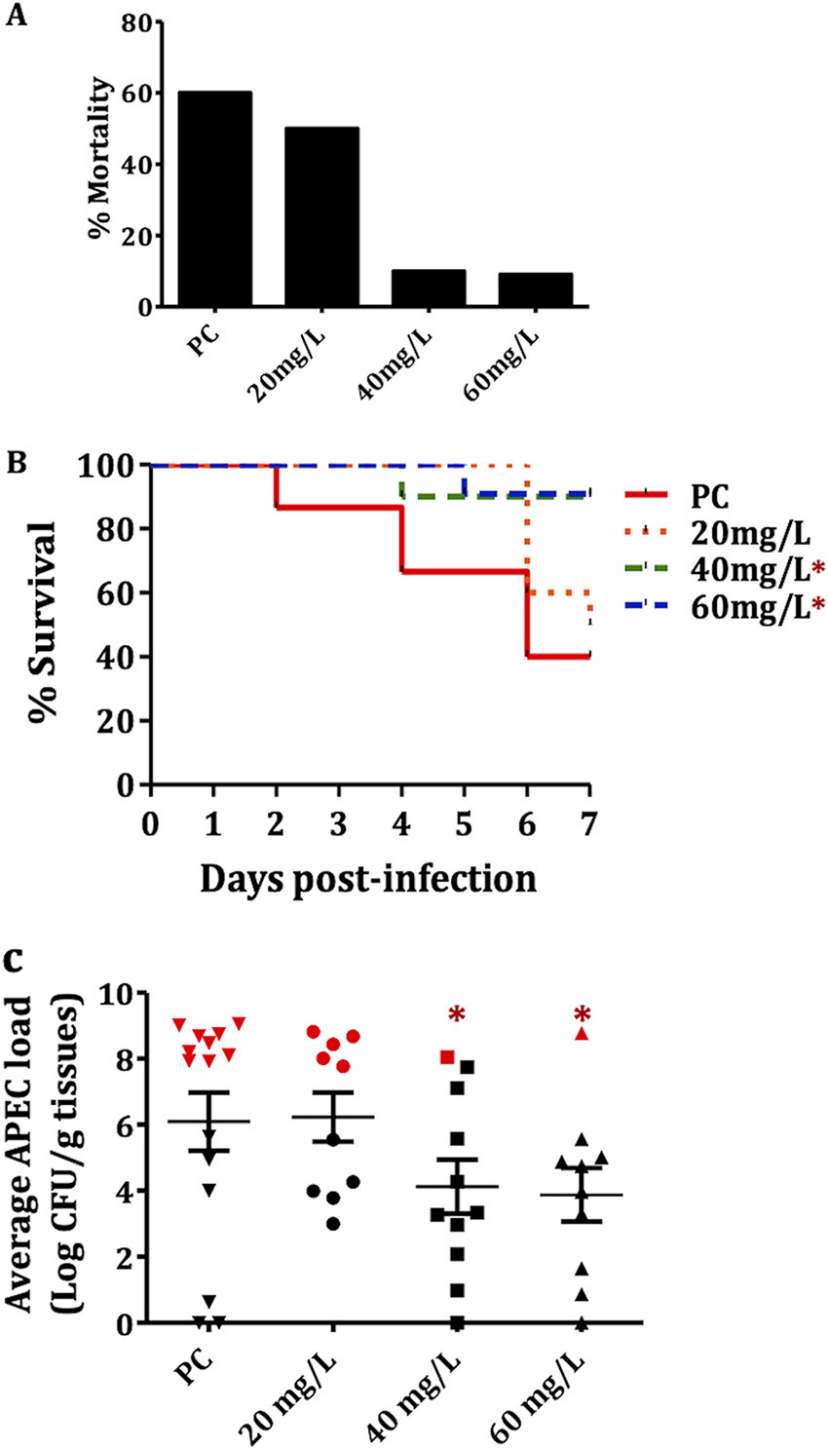
(A) Mortality (%) in GI-7 20-mg/liter, GI-7 40-mg/liter, and GI-7 60-mg/liter treatment groups compared to PC (infected and vehicle-treated; positive control) group. (B) Survival curve of chickens in PC and GI-7 20-mg/liter, GI-7 40-mg/liter, and GI-7 60-mg/liter treatment groups. *, *P* < 0.05; log-rank test. (C) APEC loads in PC and GI-7 20-mg/liter, GI-7 40-mg/liter, and GI-7 60-mg/liter treatment groups. APEC loads were quantified in internal organs (liver, heart, lung and kidney) of chickens and are displayed as the mean of all organs in each chicken. Red symbols show APEC loads in chickens that died within 7 days postinfection (dpi), and black symbols show APEC loads in live chickens sacrificed and necropsied at 7 dpi. *, *P* < 0.05; Student’s *t* test.

Since GI-7 was the most effective GI when administered orally in chickens, we selected GI-7 for further evaluation. To simulate the current field practice in the poultry industry, GI-7 was administered in drinking water for 7 days at doses of 20 mg/liter, 40 mg/liter, and 60 mg/liter. GI-7 treatments reduced the mortality by 10% to 50.9%, based on the dose, compared to that in the PC (infected and vehicle-treated; 60% mortality) group (Fig. 4A). As described above, the mortality rates observed in GI-7-treated groups were subtracted from that of the PC group. The GI-7 60-mg/liter treatment reduced the mortality by 50.9%, whereas the GI-7 40-mg/liter and GI-7 20-mg/liter treatments reduced the mortality by 50% and 10%, respectively. Furthermore, the GI-7 40-mg/liter and GI-7 60-mg/liter treatments significantly (*P* < 0.05) prolonged the survival of chickens (Fig. 4B).

Treatments with GI-7 (40 mg/liter and 60 mg/liter) reduced the severity of APEC lesions in the internal organs of chickens by up to 36.3% compared to the severity of APEC lesions in the PC group (infected and vehicle-treated) (Table 1). The PC chickens had a cumulative mean lesion score of 8.8, whereas the GI-7 60 mg/liter-treated chickens had a cumulative mean lesion score of 6.6. GI-7 40 mg/liter- and GI-7 20 mg/liter-treated chickens had cumulative mean lesion scores of 7.7 and 8.8, respectively. The highest reductions in lesion severity were observed in the GI-7 60 mg/liter-treated group (13.6% to 36.3%) (Table 1). GI-7 60-mg/liter treatment reduced the severity of liver lesions by 13.6%, heart lesions by 15.2%, lung lesions by 36.3% (*P* < 0.05), and air sac lesions by 33.8% (*P* < 0.05) (Table 1).

Treatments with GI-7 (40 mg/liter and 60 mg/liter) significantly (*P* < 0.05) reduced the APEC loads in the different internal organs of chickens, by 1.6 to 2.5 log (Fig. 4C and Table S4). GI-7 60-mg/liter treatment significantly (*P* < 0.05) reduced the APEC load in the liver by 2.4 log, in the lung by 1.6 log, in the heart by 2.4 log, and in the kidney by 2.5 log (Table S4).

A follow-up study conducted using 80 mg/liter of GI-7 did not result in a better anti-APEC effect in chickens compared to the effect of treatment at 60 mg/liter (data not shown); therefore, 60 mg/liter was used for further studies.

### GI-7 showed efficacy comparable to that of currently used antibiotic SDM without affecting BWG and feed conversion ratio (FCR) in chickens.

**FIG 5:**
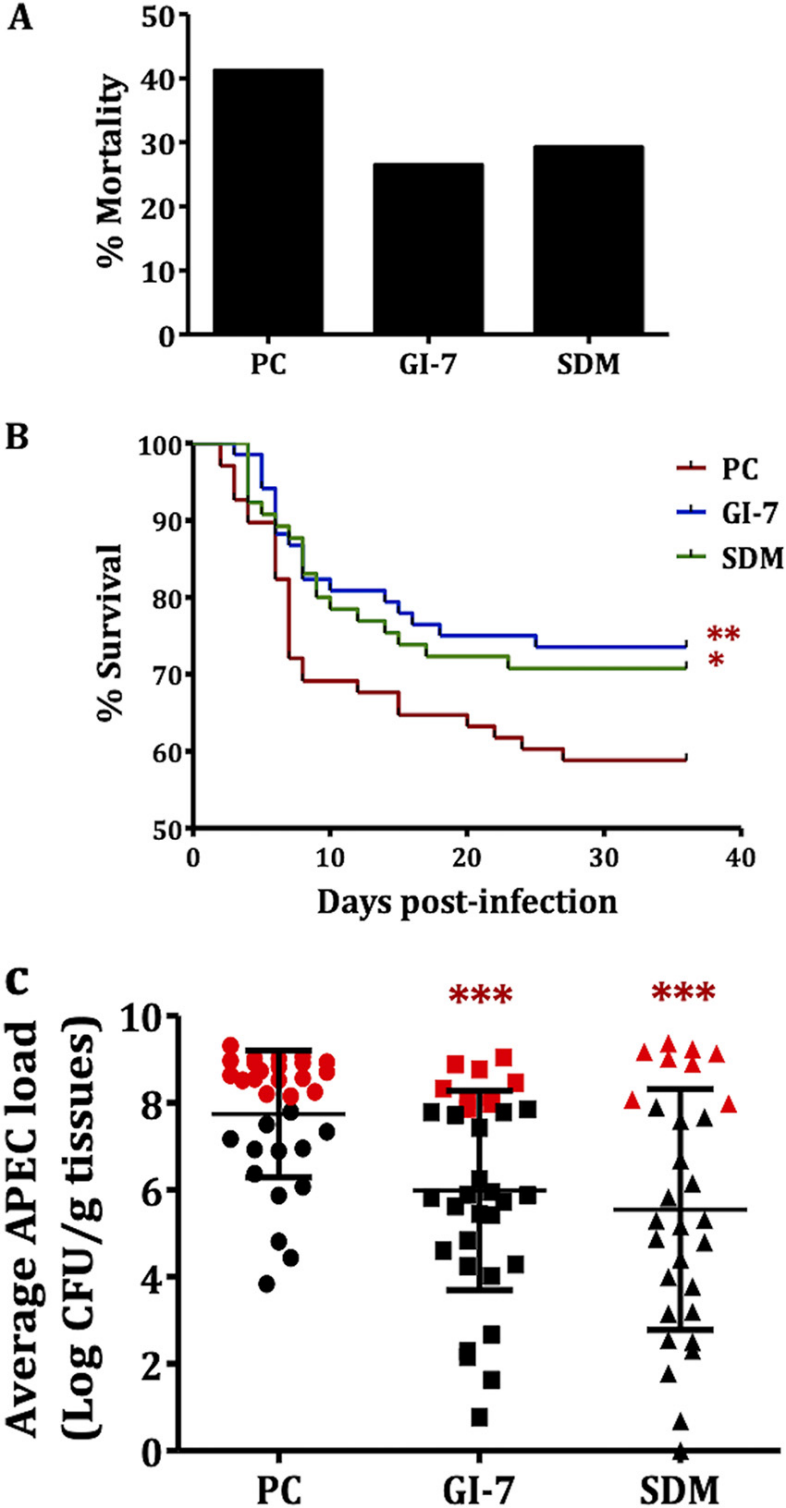
(A) Mortality (%) in GI-7- and sulfadimethoxine (SDM)-treated groups compared to PC (infected and vehicle-treated; positive control) group. (B) Survival curve of chickens in GI-7- and SDM-treated groups compared to PC group. *, *P* < 0.1; **, *P* < 0.05; log-rank test. (C) APEC loads in PC, GI-7-treated, and SDM-treated groups. APEC loads were quantified in internal organs (liver, heart, lung, and kidney) of chickens and are displayed as the mean of all organs in each chicken. Red symbols show APEC loads in chickens that died within 7 days postinfection (dpi), and black symbols show APEC loads in live chickens sacrificed and necropsied at 7 dpi. ***, *P* < 0.001; Student’s *t* test.

We compared the efficacy of GI-7 with that of sulfadimethoxine (SDM; a currently used antibiotic) in chickens under conditions simulating field conditions. GI-7 was administered at 60 mg/liter (optimized dose), whereas SDM was administered at its therapeutic dose (0.05%; 495.3 mg/liter). GI-7 reduced the mortality of chickens by 14.7% and SDM treatment reduced the mortality by 11.9% compared to that of the PC (infected and vehicle-treated; 41.2% mortality) group (Fig. 5A). As described above, the mortality rates observed in GI-7- and SDM-treated groups were subtracted from the mortality rate of the PC group. Both GI-7 and SDM treatments significantly (*P* < 0.1) increased the survival of chickens (Fig. 5B).

GI-7 and SDM treatments also significantly (*P* < 0.001) reduced the APEC load at 7 days postinfection (dpi) in the internal organs of chickens, by 1.6 to 2.5 log (GI-7, 1.6 to 2.0 log; SDM, 1.9 to 2.5 log) (Fig. 5C and Table S4). No significant difference in the APEC loads was observed between GI-7 and SDM treatments. Similarly, GI-7 and SDM treatments also decreased the severity of APEC lesions in the internal organs of chickens at 7 dpi (Table 1). The PC chickens had a cumulative mean lesion score of 11.6, whereas the GI-7- and SDM-treated chickens had cumulative mean lesion scores of 9.8 and 8.5, respectively. The reductions in lesion severity ranged from 7.2% to 29.5% in the GI-7-treated group and from 19.2% to 36.9% in SDM-treated group (Table 1). No significant difference in the APEC lesions was observed between GI-7 and SDM treatments.

No significant effect on the body weight gain (BWG) and feed conversion ratio (FCR) was observed with GI-7 and SDM treatments (Fig. S5A), which corroborates our earlier findings that GI-7 is minimally toxic to eukaryotic cells and wax moth larvae (18). Overall BWGs of 2,432.4, 2,408.1, 2,312.8, and 2,501.2 g were obtained in the GI-7, SDM, PC (infected and vehicle-treated), and NC (noninfected and not-treated) groups, respectively. Similarly, overall FCRs of 1.45, 1.49, 1.51, and 1.45 were obtained in the GI-7, SDM, PC, and NC groups, respectively (Fig. S5B).

### GI-7 was rapidly absorbed upon oral delivery in chickens, with minimal accumulation in tissues.

**FIG 6:**
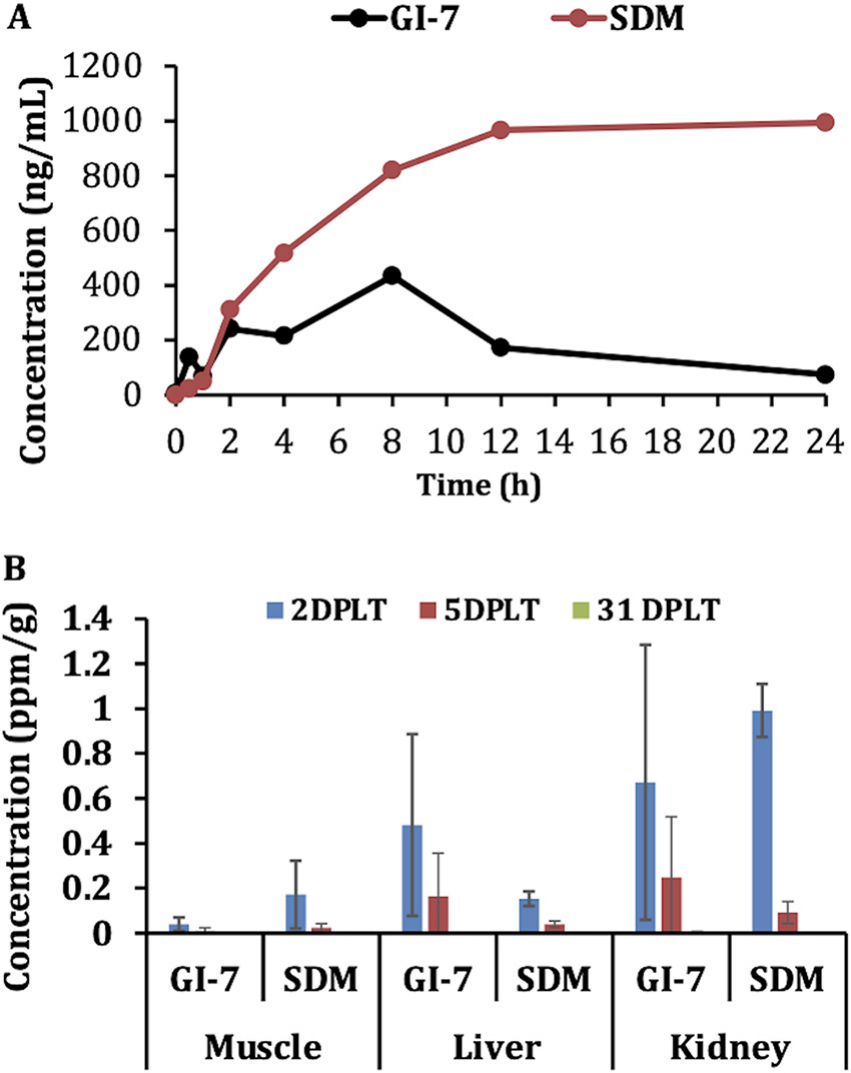
(A) Pharmacokinetic profile showing concentrations (ng/ml) of GI-7 and SDM in plasma of chickens at different time points (0, 0.5, 1, 2, 4, 8, 12, and 24 h) postadministration. (B) Concentrations (ppm/g) of GI-7 and SDM residues in tissues (muscle, liver, and kidney) of chickens. DPLT, days post-last treatment; SDM, sulfadimethoxine.

Pharmacokinetic (PK) analysis of the plasma of GI-7-treated chickens revealed that GI-7 is absorbed rapidly (0.5 h) compared to the rate of absorption of sulfadimethoxine (SDM) (2 h) (Fig. 6A). Similarly, GI-7 is excreted by 24 h, whereas no excretion of SDM was observed until 24 h. The peak plasma concentration (*C*_max_, 434.2 ± 388.5 ng/ml [mean ± standard deviation]) of GI-7 was reached at 8 h (time to maximum concentration [*T*_max_]) postadministration, while the *C*_max_ and *T*_max_ for SDM were 993.6 ± 166.9 ng/ml and 24 h, respectively. Interestingly, no cumulative accumulation of GI-7 in plasma was observed after repeated administration, whereas accumulation was observed with SDM (data not shown). These data indicate that GI-7 has a shorter half-life than SDM.

The residue analysis of GI-7 in chicken tissues showed relatively less or no accumulation of GI-7 compared to the accumulation of SDM, except in the liver (Fig. 6B). In the muscle, concentrations of 0.04 ± 0.03, 0.01 ± 0.01, and 0.0 ± 0.0 ppm/g tissue of GI-7 were observed at 2, 5, and 31 days post-last treatment (DPLT), respectively, while concentrations of 0.2 ± 0.1, 0.02 ± 0.02, and 0.0 ± 0.0 ppm/g tissue of SDM were observed at 2, 5, and 31 DPLT, respectively. Similarly, in the kidney, residue levels of 0.7 ± 0.6, 0.3 ± 0.3, and 0.0 ± 0.0 ppm/g tissue were observed in GI-7-treated chickens at 2, 5, and 31 DPLT, respectively, while residue levels of 1.0 ± 0.2, 1 ± 0.1, and 0.0 ± 0.0 ppm/g tissue of SDM were observed at 2, 5, and 31 DPLT, respectively. On the other hand, in the liver, residue levels of 0.5 ± 0.4, 0.2 ± 0.2, and 0.0 ± 0.0 ppm/g tissue of GI-7 were observed at 2, 5, and 31 DPLT, respectively, while residue levels of 0.2 ± 0.04, 0.04 ± 0.02, and 0.0 ± 0.0 ppm/g tissue of SDM were observed at 2, 5, and 31 DPLT, respectively. The residue quantitation of GI-7 in tissues of chickens showed GI-7 residues below the limits permitted by the Food and Drug Administration for antibiotics (35), which ensures the safety of GI-7 from the public health standpoint (36).

### Expression of genes essential for OM integrity was downregulated and levels of Lpt proteins were decreased in GI-7-treated APEC.

**FIG 7:**
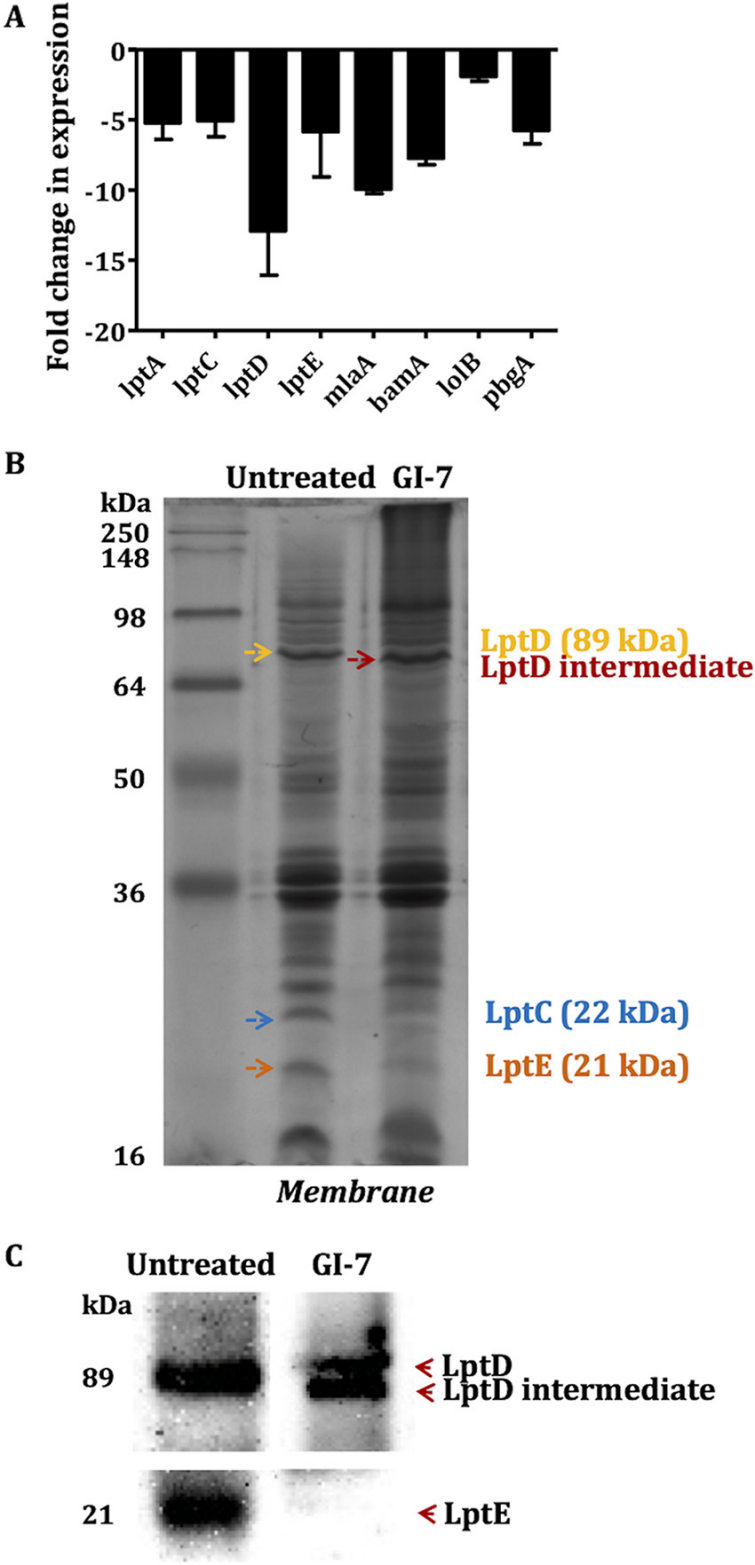
(A) Effects of GI-7 treatment on the expression of *lpt* and other genes essential for maintaining outer membrane (OM) integrity as determined by RT-qPCR. Fold change was calculated using the ΔΔ*C_T_* method. (B) Effects of GI-7 treatment on total membrane proteins as determined by SDS-PAGE and staining with Coomassie blue R-250. Yellow arrow shows expected size of LptD, red arrow shows potential LptD intermediate, orange arrow shows expected size of LptE, and blue arrow shows expected size of LptC. (C) LptD and LptE immunoblots performed using anti-LptD and anti-LptE antibodies showing formation of potential LptD intermediate and depleted LptE level in GI-7-treated APEC compared to untreated APEC.

Based on the similarity in BCPs (bacterial cytological profiles; accumulation of membrane-like material in the form of knobs inside the cells) observed between GI-7 (18), thanatin, and JB-95 (reported Lpt inhibitors) (22, 23), we investigated the effect of GI-7 on the expression of *lpt* genes by reverse transcription-quantitative PCR (RT-qPCR) and on total membrane proteins by SDS-PAGE (under nonreducing conditions) followed by liquid chromatography-tandem mass spectrometry (LC-MS/MS) analysis of the excised ∼21.4-kDa (expected size of LptE protein) gel fragments. GI-7 treatment downregulated the expression of the *lptD* gene by 12.90-fold, while the *lptE*, *lptA*, and *lptC* genes were downregulated by 5.39-, 5.13-, and 4.99-fold, respectively, compared to their expression in untreated APEC (Fig. 7A). Furthermore, downregulation of *lptD* was higher than that of other OM genes (*bamA*, *lolB*, *mlaA*, and *pbgA*). Downregulation of expression by 7.72-, 1.92-, 9.94-, and 5.75-fold was observed for genes *bamA*, *lolB*, *mlaA*, and *pbgA*, respectively. Furthermore, in SDS-PAGE (nonreducing conditions), a decreased level of an ∼21.4-kDa (expected size of LptE) protein was observed with GI-7 treatment (Fig. 7B). Additionally, slightly faster mobility of an approximately 89-kDa protein (expected size of LptD) was observed in GI-7-treated APEC compared to its mobility in untreated APEC (Fig. 7B), suggesting the possible formation of an LptD intermediate (or nonfunctional LptD). The LptD intermediate with slightly faster mobility was observed in E. coli when LptE was limited (37). LC-MS/MS analysis also showed a depleted level (or low exponentially modified protein abundance index [emPAI]) of LptE in GI-7-treated APEC (emPAI of 8.46) compared to the level (emPAI of 13.84) in untreated APEC. Furthermore, immunoblot studies performed using anti-LptD and anti-LptE antibodies (GenScript) showed that GI-7 treatment decreased the LptE level and showed LptD with slightly faster mobility compared to that in untreated APEC, which might represent a nonfunctional LptD intermediate, as previously observed when LptE is limited (Fig. 7C) (37). A decreased level of another protein, of ∼22 kDa, which is the expected size of LptC (21.7 kDa), was also observed with GI-7 treatment (Fig. 7B). No noticeable changes in the cytoplasmic protein profiles were observed between GI-7-treated and untreated APEC in SDS-PAGE (data not shown).

### GI-7 decreases OM LPS level and is predicted to interact with Tyr244, Lys234, and Glu733 residues of LptD.

**FIG 8:**
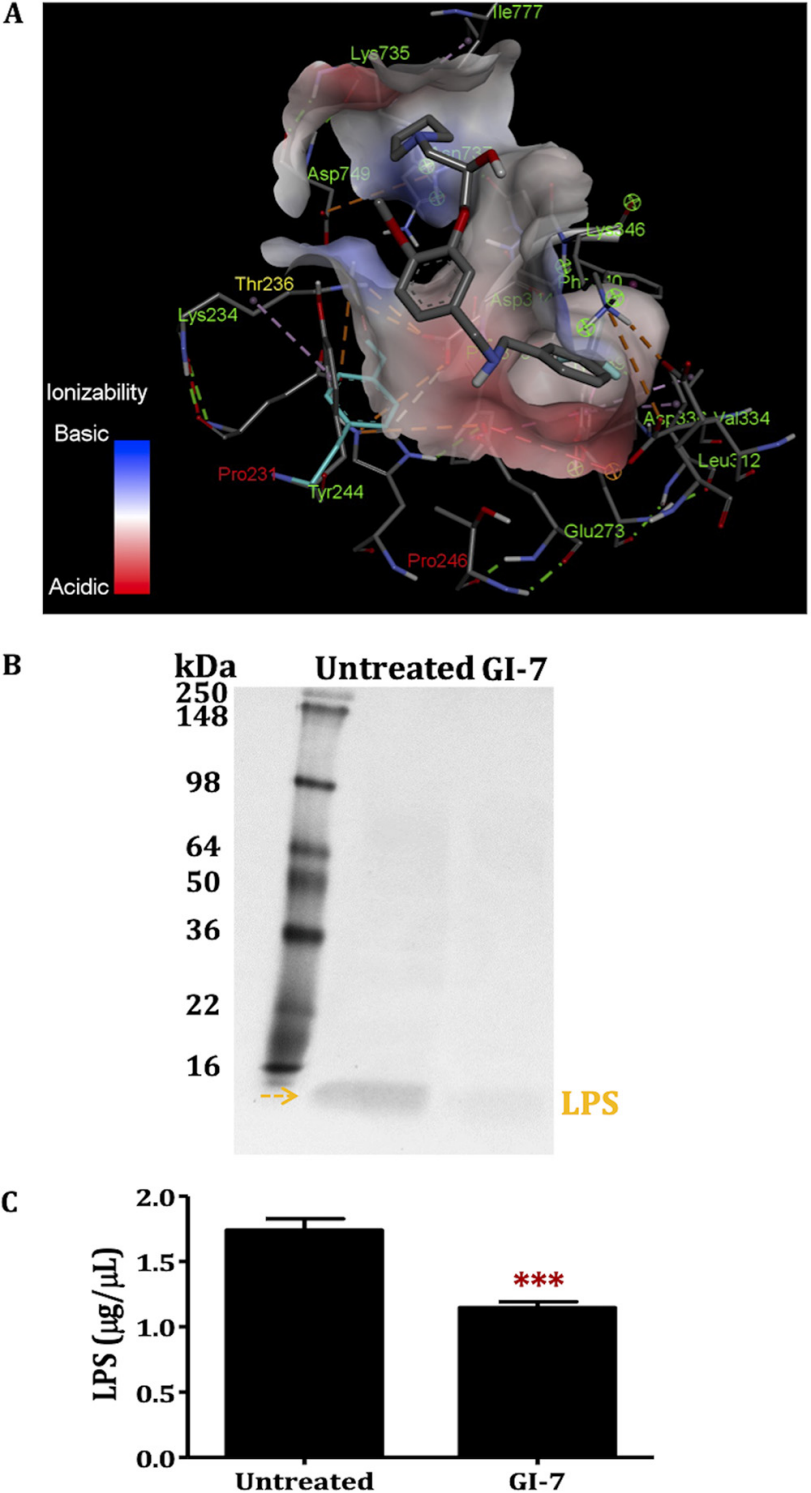
(A) Predicted binding mode of GI-7 in the pocket of LptD. GI-7 is predicted to interact with key amino acid residues (i) Tyr244, which is flanked by Pro231 and Pro246 (shown in red) at the lateral gate of LptD, and (ii) Lys234, which is next to Thr236 (shown in yellow) inside the lumen of LptD. (B and C) Effect of GI-7 treatment on the level of outer membrane lipopolysaccharide (LPS; yellow arrow) as visualized by Coomassie staining (B) and quantitated by a total carbohydrate assay kit (C). ***, *P* < 0.0001; Student’s *t* test.

*In silico* docking studies using Autodock predicted that GI-7 is likely to bind in the LptD pocket between the β1 and β26 strands, wherein LPS enters the lumen channel (Fig. 8A and Fig. S6A) (38). GI-7 is predicted to interact with key amino acid residues (i) Tyr244, which is flanked by Pro246 and Pro231 at the lateral gate of the lumen, and (ii) Lys234, which is next to Thr236 inside the lumen of LptD (Fig. 8A and Fig. S6B and C). Additionally, docking using Autodock Vina predicted that GI-7 is also likely to bind with Glu733, in addition to Tyr244 and Lys234 (Fig. S7A). The predicted hydrogen bonding interactions of GI-7 with the Tyr244 and Lys234 residues of LptD are portrayed in Fig. S7B and C. The docking of GI-2 and GI-3, which have structures similar to that of GI-7 (Fig. 1A) but are less effective against APEC (18), revealed lower predicted binding affinities of GI-2 (−6.75 kcal/mol) and GI-3 (−7.26 kcal/mol) with LptD compared to that of GI-7 (−9.05 kcal/mol) (Table S7). Furthermore, the binding affinities of GI-6 (−4.75 kcal/mol) and GI-10 (−5.61 kcal/mol), which have different structures (Fig. 1A) than GI-7, are even lower than those of GI-2 and GI-3, suggesting that GI-7 and other compounds with structural similarity to GI-7 might specifically bind to LptD.

To further support that GI-7 affects the Lpt levels and, thereby, likely the LPS transport, we determined the LPS level in the OM of APEC treated with GI-7. A lower level of LPS was observed in OM of APEC treated with GI-7 than in OM of untreated APEC (Fig. 8B). This was further confirmed by quantitating the LPS levels using the total carbohydrate assay kit, which revealed a significantly (*P* < 0.0001) lower level of LPS in OM of GI-7-treated APEC than in OM of untreated APEC (Fig. 8C).

## DISCUSSION

Previously, we identified eight small molecule growth inhibitors (GIs) (Fig. 1A) with promising *in vitro* (epithelial, macrophage, and red blood cells) and *in vivo* (wax moth larva model) activities against APEC (18). In this study, we show three GIs containing pyrrolidinyl (GI-7), nitrophenyl (GI-10), and imidazole (GI-6) scaffolds as having protective effects against APEC infection in chickens (Fig. 1 and 2). Among the three GIs, GI-7 was the most effective when administered orally and also showed efficacy when delivered in drinking water at a 60-mg/liter dose (Fig. 4 and 5), a dose 8 times lower than the standard dose of sulfadimethoxine (SDM), an antibiotic currently used to treat APEC infection in chickens. The delivery of GI-7 in drinking water simulates the current antibiotic treatment practice for treating APEC infection in the poultry industry (14). Notably, the dose of GI-7 (60 mg/liter) is relatively lower (3 to 75 times) than the doses of antibiotics commonly used in the poultry industry (sulfadimethoxine, 495 mg/liter; sulfaquinoxaline, 200 mg/liter; chlortetracycline, 4.5 g/liter; and ampicillin, 1.65 g/liter) (14). This has a significant advantage from the perspective of treatment costs, as well as the accumulation of drug residues in tissues of chickens, a parameter essential for the safety of food for human consumption (36). Furthermore, no *in vivo* resistance to GI-7 was detected in APEC isolated from chickens treated with GI-7. Moreover, resistance was also not detected *in vitro* in our earlier study when APEC was incubated with a lethal concentration or passaged with a sublethal concentration of GI-7 (18). Our findings are therefore noteworthy in the current situation, where high levels of resistance to available antibiotics have been reported (17) and there are increased restrictions on the use of medically important antibiotics in food-producing animals (39). The lack of APEC resistance to GI-7 might be due to the membrane-targeting mechanism of GI-7 (18), although a more thorough search for and characterization of GI-7-resistant mutants is necessary to facilitate industrial application of GI-7 as an anti-APEC therapeutic in the future. Membrane-targeting antibacterial drugs are less likely to acquire resistance and are also effective against antibiotic-resistant strains (20, 40). The APEC strain used in our chicken studies is resistant to ampicillin and tetracycline, two of the antibiotics most commonly used in food animals (18). Furthermore, our earlier study also showed that GI-7 can potentiate the effects of other antibiotics (colistin, tetracycline, and ciprofloxacin) (41) and, thus, can synergistically enhance the efficacy of current antibiotics. Therefore, combination therapy with GI-7 has a great potential to lower the dose of antibiotics needed for the treatment, which thereby can mitigate the emergence of antibiotic resistance (42) and potential transmission of antibiotic resistance genes (ARGs) to humans (43).

Studies including bacterial cytological profiling (18), expression of *lpt* genes, LptD/E immunoblot analysis, LPS quantitation in the OM, and *in silico* docking were performed to explore the potential mechanism of GI-7 (Fig. 7 and 8 and Fig. S6 and S7). GI-7 affected the OM integrity and decreased the Lpt protein and LPS levels in APEC. LPS is critical for bacterial growth, virulence, survival, and maintenance of the OM permeability barrier (44). Furthermore, LPS is an essential component for OM biogenesis/assembly in Gram-negative bacteria (44). The altered LPS level compromises the OM permeability barrier, thus resulting in loss of bacterial viability and growth (37). The OM permeability barrier is also responsible for inducing bacterial resistance against antibiotics by hindering antibiotics from reaching their intracellular targets (19, 21). Moreover, LPS plays a key role in virulence and pathogenesis of bacteria in the host (45, 46). Therefore, the LPS transport pathway is regarded as a novel and attractive target for the development of new antibacterial drugs, as well as to counter antibiotic resistance in Gram-negative pathogens (19, 21). LPS transport from the IM to the OM is mediated by the Lpt complex (LptABCDEFG) (37, 47). LptB_2_FGC transports LPS from the IM to the periplasmic space, followed by transport across the periplasmic space by LptA and, finally, to the OM by LptD/E (37). The interaction of LptD with LptE (i.e., the LptD/E complex) is crucial for the transport of LPS to the OM in Gram-negative pathogens (48). Our studies show that GI-7 treatment might induce the formation of LptD intermediate, as shown by slightly faster migration than for mature wild-type LptD in SDS-PAGE and an additional LptD band (or new species of LptD) in immunoblot analysis (37). The formation of LptD intermediate was also reported previously when LptE was depleted, similar to what we observed in our study, or in LptD mutants (LptDΔ330-352 and LptDΔ529-538) (37). LptE is essential for the proper assembly and maturation of functional LptD with the proper arrangement of disulfide bonds (37, 46, 49). The LptD intermediate, which lacks native disulfide bond(s), is nonfunctional and incapable of transporting LPS from the periplasmic space to the OM, resulting in defective bacterial OM (37, 49). The nonfunctional LptD intermediate is formed when LptE is limited and results in depleted LPS levels in the OM (37, 49). We showed a lower level of LPS in the OM of APEC treated with GI-7, in support of decreased Lpt protein levels in APEC treated with GI-7. Based on these data, a hypothetical mechanism of action (MOA) of GI-7 is displayed in Fig. S8. However, future studies that incorporate control antibiotics, including antibiotics already known to act through the Lpt system (for example, murepavadin) and antibiotics affecting OM integrity without interacting with the Lpt system (for example, colistin), are necessary to validate the Lpt system as a target of GI-7. Furthermore, the effect of GI-7 on the Lpt system could be indirect or secondary to other mechanism(s) of GI-7. Therefore, further investigation through direct GI-7–LptD/E binding studies using biophysical methods like nuclear magnetic resonance (NMR) and X-ray crystallography (XRC) are needed to validate GI-7’s interaction with the Lpt system. It is also important to recognize that GI-7 has an antibacterial effect on Gram-positive bacteria as well (18), suggesting that it might also function through other targets, characteristic of membrane-acting antibacterials (40). Therefore, a comprehensive analysis is needed to better define the MOA(s) of GI-7. In fact, a preliminary thermal proteome profile (TPP) (50) of GI-7-treated APEC indicated LptD/E as a potential target of GI-7; however, multiple other proteins with increased abundance were also identified, suggesting potential interaction of GI-7 with other targets (data not shown).

Docking studies predicted that GI-7 is likely to interact with LptD (Fig. 8 and Fig. S6 and S7). Recently, many antibacterials targeting the Lpt complex, especially LptD and LptA, have been identified (22–25, 51). Murepavadin and L27-11, the peptidomimetic antibiotics effective against MDR Pseudomonas, bind to the periplasmic segment of LptD and block LPS transport to the OM (24, 25). Similarly, JB-95, a macrocyclic peptide highly effective against MDR E. coli, downregulates LptD, thus affecting OM biogenesis (22). Likewise, thanatin, an insect-derived antimicrobial peptide, binds to LptA and LptD, which leads to inhibition of LPS transport and, subsequently, OM assembly in E. coli (23). GI-7 was also effective against antibiotic-resistant APEC strains (18), which supports LptD as the potential target for its antibacterial action. Resistance mechanisms against LptD inhibitors are not widely reported, although a recent report has suggested that mutations in *pmrB* can confer cross-resistance against an LptD inhibitor, POL7080, in P. aeruginosa (52). Our study suggests that LptD/E is a valuable druggable target, and confirmation of the direct interaction of GI-7 with LptD/E using biophysical methods like surface plasmon resonance (SPR) and isothermal titration calorimetry (ITC), as well as site-directed mutagenesis studies, will pave the way for the development of novel and effective antibacterial drugs. The downregulation of expression of other OM genes, particularly *bamA* and *mlaA*, might be an off-target/secondary effect of *lptD* inhibition by GI-7. Decreased levels of BamA and multiple other OM proteins were also observed with JB-95 (LptD inhibitor) treatment in E. coli (22). Similarly, downregulation of MlaA and LptD proteins was observed concurrently in LPS-deficient Acinetobacter baumannii (53).

GI-7 did not significantly alter the microbial composition (at the phylum level) of the cecum of chickens (Fig. 3A), which might suggest a minimal impact on the host microbiota (54). Furthermore, the increased *Lactobacillus* (L. zeae and L. helveticus) population in GI-7-treated chickens (Fig. 3B and Table 2) might be responsible for the better anti-APEC activity of GI-7 compared to that of other GIs. The antibacterial effects of L. zeae and L. helveticus have been reported previously (55–59). Pretreatment with L. zeae strain LB1 protected Caenorhabditis elegans from enterotoxigenic E. coli (ETEC) infection by upregulating the production of antimicrobial peptides and host defense molecules (55) and inhibiting the enterotoxin expression (56). Similarly, feeding fermented feed containing L. zeae reduced the Salmonella enterica serovar Typhimurium strain DT104 burden in experimentally challenged weaned pigs (57). Likewise, L. helveticus strain SP-27 reduced *S.* Typhimurium infection in human patients (58) and prevented the death of neonatal mice caused by Citrobacter rodentium infection (59). GI-7 also did not significantly alter the serum metabolites (Table 3 and Fig. S3), which might be due to the minimal impact of GI-7 on the cecal microbiota of chickens (60). Oleate is the major serum metabolite elevated in chickens treated with GI-7 (Table 3). Oleate is required for the induction of innate immune defenses and protection against diverse bacterial pathogens (P. aeruginosa, Enterococcus faecalis, and Serratia marcescens) in C. elegans (61). Furthermore, oleate possesses significant antibacterial activity against methicillin-resistant Staphylococcus aureus (MRSA), Helicobacter pylori, and *Mycobacteria* (62). Oleate interferes with fatty acid synthesis through inhibition of the bacterial enoyl-acyl carrier protein reductase (FabI) (62).

In our study, we infected the chickens with 10^7^ CFU of APEC using the subcutaneous (s.c.) APEC infection model, which is a reproducible and commonly used acute infection model causing higher mortality and rapid progression of disease in chickens (63). However, under field conditions, chickens usually get exposed to lower APEC levels (10^2^ to 10^3^ CFU) through oral or aerosol routes and the infection progresses relatively more slowly (10, 63). Furthermore, the presence of concurrent viral (Newcastle, infectious bronchitis, and avian influenza virus) and *Mycoplasma* infections and stress (high CO_2_, NH_3_, and humidity) predispose the chickens to APEC infection (10). Therefore, future investigations involving the natural routes of APEC infection or naturally infected chickens will be an important consideration to advance the development of GI-7 for therapeutic application in poultry farms. Approaches complementary to GI-7 might be advantageous for the complete protection of chickens against APEC. Recent studies have reported the potential use of immunomodulators like Toll-like receptor (TLR) agonists, NOD-like receptor agonists (NLR), innate defense regulator (IDR) peptides, probiotics, and surfactins as adjuvants for antibacterial therapy (64–67). GI-7 can also be combined with antibiotics currently being used in the poultry industry to enhance colibacillosis control, since GI-7 showed synergistic activity with different antibiotics (e.g., colistin, tetracycline, and ciprofloxacin) in our earlier study (41). Additionally, in our separate studies, we have identified beneficial bacteria and peptides (68) and APEC virulence inhibitors (33) displaying anti-APEC activity, which can be combined to provide better protection against APEC infection in chickens.

In summary, this study identified a promising novel small molecule growth inhibitor, GI-7, effective against APEC infection in chickens. Our studies showed that GI-7 affected the OM and decreased the Lpt protein and LPS levels in APEC. Antibacterial drugs targeting the bacterial OM can overcome the resistance problem, and thus, the OM represents a promising target for novel antibacterial development against Gram-negative pathogens. Furthermore, with APEC being genetically similar to human ExPECs, our findings can have profound implications in developing new antibacterial drugs against human ExPECs. Our future studies will focus on improving the efficacy of GI-7 using medicinal chemistry methods. We will also validate the Lpt system (LptD/E) as a target of GI-7 by using biophysical methods to measure the binding affinity of GI-7 to LptD/E proteins and site-directed mutagenesis studies to determine the essentiality of LptD residues predicted to bind with GI-7.

## MATERIALS AND METHODS

### Ethics in animal experimentation.

All chicken experiments conducted in this study were approved by The Ohio State University Institutional Animal Care and Use Committee (IACUC) (protocol number 2010A00000149). Chickens were euthanized using CO_2_ following American Veterinary Medical Association (AVMA) guidelines. Approved husbandry practices were followed throughout the experiments.

### Efficacy of GIs against APEC infection in chickens.

The efficacy of GIs was assessed using commercial broiler chickens (*n* = 6/group). A total of eight GIs (GI-1, GI-2, GI-3, GI-6, GI-7, GI-8, GI-9, and GI-10) (Fig. 1A) identified in our previous high-throughput study (18) were tested. The detailed experimental approach is displayed in Fig. S1A. GI compounds (ChemBridge, San Diego, CA) dissolved in dimethyl sulfoxide (DMSO) were administered orally using a syringe, twice a day, from day 4 (1 day before APEC infection) to day 8 (3 days postinfection [dpi]). The dose of each GI is described in Table S1. The doses corresponded to 50× the *in vitro* minimum bactericidal concentrations (MBCs) of the GIs and were selected based on previous studies (69, 70). Chickens that were infected and vehicle-treated (positive control [PC]) and noninfected and nontreated (negative control [NC]) were included as controls. On day 5, chickens were infected subcutaneously (s.c.) with rifampin-resistant (Rif^r^) APEC serotype O78 (18) (1 × 10^7^ CFU/chicken). This dose, which resulted in consistent productive infection, was selected based on a preliminary study with different infection routes (s.c., intratracheal, and intra-airsac) and doses (10^6^, 10^7^, and 10^8^ CFU/chicken) to determine the appropriate route and dose for establishment of APEC infection in chickens (Table S2). To prepare the APEC inoculum for infection, Rif^r^ APEC O78 (50 μg/ml rifampin) was grown to logarithmic phase in LB medium, washed twice with phosphate-buffered saline (PBS), and adjusted to an optical density at 600 nm (OD_600_) of 0.1 (5 × 10^7^ CFU/ml). The clinical signs and mortality were recorded until 7 dpi. Chickens that died during this period were necropsied on the same day, lesions in internal organs (liver, heart, lung, and air sacs) were scored as described previously (63, 71), and the APEC loads were quantified in internal organs (liver, heart, lung, and kidney) by plating the tissue homogenates on MacConkey agar plates containing 50 μg/ml rifampin (18). At 7 dpi (day 12), all live birds were euthanized, lesions were scored, and the APEC loads in internal organs were quantified as described above. The body weight of chickens was measured before GI treatment (day 3) and at the day of necropsy (day 12).

### Evaluation of resistance acquisition *in vivo*.

To test whether APEC developed resistance against GIs (GI-7, GI-10, and GI-6) posttreatment in chickens, APEC colonies (*n* = 24/GI-treated group) cultured from different organs were randomly selected from the agar plates at the end of the experiments, grown in M63 minimal medium (37°C and 200 rpm), and tested for growth inhibition by GIs at their respective MICs and MBCs as described previously (18).

### Effect of GIs on cecal microbiota of chickens.

To investigate the impact of GIs on the cecal microbiota of chickens, a 16S rRNA-based metagenomic study was conducted as described previously (69, 72). Cecal microbiota was analyzed for only those GIs (GI-7, GI-10, and GI-6) that had an impact on APEC infection in the experiment described above. For the metagenomic analysis, the QIIME 2 (Quantitative Insights Into Microbial Ecology 2) bioinformatics platform (73, 74) was used. Details of methods are included in the supplemental material. The statistical difference (*P* < 0.05) in the taxonomic composition between different groups was determined using the Mann-Whitney U test. The alpha and beta diversity were analyzed using the Kruskal-Wallis test and permutational multivariate analysis of variance (PERMANOVA) (*P* < 0.05), respectively.

### Effect of GIs on serum metabolome of chickens.

To investigate the impact of GIs on the serum metabolites of chickens, the untargeted serum metabolome profile was determined using liquid chromatography-mass spectrometry (LC-MS) at the Campus Chemical Instrumentation Center, Mass Spectrometry and Proteomics Facility, The Ohio State University (CCIC, MS&P Facility, OSU) (https://live-ccic.pantheonsite.io/MSP). Metabolome profiling was performed on a Thermo Orbitrap LTQ XL instrument in positive-mode analysis with high-performance liquid chromatography (HPLC) separation on a Thermo Scientific RLCS ultimate 3000 LC system using a Poroshell 120 SB-C_18_ (2 by 100 mm, 2.7-μm particle size) column. The significantly (*P* < 0.05, Kruskal-Wallis test) altered metabolites and the associated metabolic pathways were identified using XCMS Online (https://xcmsonline.scripps.edu/) and expressed as the fold changes in abundance compared to the abundance in NC (noninfected and nontreated) chickens. Principal-component analysis (PCA) was performed to compare the metabolome profiles between the treatment groups. Progenesis QI software (Waters/Nonlinear Dynamics) was used to validate the results. Details of methods are included in the supplemental material.

### Dose optimization of GI-7 in drinking water.

Among the GIs tested, GI-7 showed the most potent anti-APEC activity in chickens when administered orally in the pilot experiment described above; therefore, we optimized the dose of GI-7 for drinking water delivery in chickens (a common industry practice). GI-7 was administered at different doses (20 mg/liter, 40 mg/liter, and 60 mg/liter), and chickens were challenged (s.c.) with Rif^r^ APEC O78 (5 × 10^6^ CFU/chicken). Prior to selection of these doses, we conducted a preliminary study using 20-mg/liter, 200-mg/liter, and 500-mg/liter doses (doses similar to those of antibiotics currently used in the poultry industry). Only 20 mg/liter showed a promising result in reducing mortality, lesions, and APEC load (data not shown), and higher doses were toxic to chickens; therefore, we tested further using 20-mg/liter, 40-mg/liter, and 60-mg/liter doses. The experiment was conducted using commercial broiler chickens (*n* = 11/group) as described above following procedures described in Fig. S1A. GI-7 was administered in drinking water containing 0.05% DMSO from day 4 to day 10 (a total of 7 days). The amount of drinking water was adjusted daily based on the age and requirement of chickens (https://www.thepoultrysite.com/articles/broiler-water-consumption). The clinical signs and mortality, lesion scores, and APEC loads were determined as described above. We also tested GI-7 at an 80-mg/liter dose in a follow-up study.

### Comparative efficacies of GI-7 and sulfadimethoxine in large chicken trial simulating field conditions (floor trial).

The optimized dose of GI-7 (60 mg/liter) and therapeutic dose of sulfadimethoxine (SDM; 0.05% or 495.3 mg/liter) were used to compare the efficacies of GI-7 and SDM in commercial broiler chickens (*n* = 70/group) raised on built-up floor litter. GI-7 and SDM were administered in drinking water for 7 consecutive days from day 5 (1 day prior to infection) to day 11 (5 dpi). The detailed experimental approach is displayed in Fig. S1B. On day 6, chickens were infected (s.c.) with Rif^r^ APEC O78 (5 × 10^6^ CFU/chicken). At day 13 (7 dpi), half of the chickens from each group, and at day 42 (slaughter age), 10 chickens from each group were necropsied and lesions and APEC loads were assessed as described above. The body weight of the chickens was measured weekly, and feed intake was recorded daily to compare the effects of GI-7 and SDM on the body weight gain (BWG) and feed conversion ratio (FCR).

### Pharmacokinetic (PK) study.

The absorption and excretion kinetics of GI-7 upon oral delivery in chickens (from experimental set-up as described for the floor trial above) were determined using LC-MS (CCIC, MS&P Facility, OSU) as described previously (75). Five chickens from each group were sacrificed at 0, 0.5, 1, 2, 4, 8, 12, 24, 84, and 180 h posttreatment, and plasma samples were mixed with cold methanol (MeOH) followed by the addition of 10 μl of 1 μg/ml internal drug standard (heavy-labeled phenylalanine dissolved in H_2_O and 0.1% formic acid [FA]). Proteins were removed by ultracentrifugation, and 5-μl amounts of supernatants were run using the Agilent Poroshell 120 SB-C_18_ column (2 by 100 mm, 2.7-μm particle size). The quantitation of GI-7 in plasma was performed by calibrating standard solutions of GI-7 containing different concentrations (0.001, 0.005, 0.01, 0.05, 0.1, 0.5, 1, and 5 μg/ml) in LC-MS analyses run as described above (Fig. S2A and B). Plasma samples from chickens treated with SDM were used for comparison. GI-7 was monitored at 439.2→232.1 *m/z* at 20 V collision energy (CE) and sulfadimethoxine at 311.1→156 *m/z* at 30 V CE. Details of the methods are included in the supplemental material.

### Quantification of GI-7 residue in tissues.

The GI-7 residue in chicken tissues (muscle, liver, and kidney) was quantified using LC-MS (CCIC, MS&P Facility, OSU) as described previously (76). Chickens (*n* = 5/time point) from an experimental set-up as described above for the floor trial were sacrificed at 2 days (day 13; 2 DPLT), 5 days (day 16; 5 DPLT), and 31 days (day 42; 31 DPLT) post-last treatment. The quantitation of GI-7 in chicken tissues was performed by calibrating against the standard solutions of GI-7 containing different concentrations (0.1, 0.5, 1, 5, 10, 50, 100, 500, and 1,000 ng/ml) (Fig. S2C and D). Tissue samples from chickens treated with SDM were used for comparison. GI-7 and SDM peaks were monitored at 439.2→232.1 *m/z* and 311.1→156 *m/z*, respectively, as described above. Details of the methods are included in the supplemental material.

### Gene expression analysis.

To understand the potential mechanism of GI-7’s antibacterial effect, the expression of *lpt* genes (*lptD*, *lptE*, *lptA*, and *lptC*) was quantitated using RT-qPCR as described previously (33, 41). These genes were selected based on the similarity of the bacterial cytological profile (BCP; accumulation of membrane-like material in the form of knobs inside the cells) induced by GI-7 (18) to the BCPs of thanatin (23) and JB-95 (22), which were identified as Lpt inhibitors in E. coli (22, 23). We also quantitated the expression of other genes essential for maintaining OM integrity (*bamA*, *mlaA*, *lolB*, and *pbgA*) (21). An APEC O78 culture grown overnight was adjusted to an OD_600_ of 0.5 and treated with GI-7 for 4 h to achieve ∼50% growth inhibition (8 replicates, 200 μl each) (Fig. S3) at 37°C with shaking at 200 rpm as previously described (22, 77, 78). An APEC O78 culture treated with 1% DMSO was used as a control. Total RNA was extracted using the RNeasy minikit (Qiagen), and 5 μg of purified RNA was used to synthesize cDNA using the RT^2^ first strand kit (Qiagen). The RT-qPCR was performed using Maxima SYBR green/ROX qPCR master mix (Thermo Fisher) in a RealPlex^2^ Mastercycler (Eppendorf) with a 55°C annealing temperature. The primers (Table S3) were designed using the PrimerQuest tool and obtained from Integrated DNA Technologies (IDT). The data were normalized to the housekeeping gene encoding glyceraldehyde-3-phosphate dehydrogenase (GAPDH), and relative fold changes were calculated using the cycle threshold (ΔΔ*C_T_*) method (79). Two independent experiments were conducted.

### Protein expression and LC-MS/MS analysis.

To support the gene expression findings, the levels of total membrane proteins upon GI-7 treatment were investigated as described previously (22). A GI-7-treated culture of APEC O78 was prepared as described above, and the fractions of cytoplasmic and total membrane proteins were prepared using ultracentrifugation as described previously (80). The concentration of fractionated proteins was normalized, and 25 μg of proteins were run on 12% SDS-PAGE under nonreducing (in the absence of β-mercaptoethanol) conditions (37). The gel was stained with Coomassie blue R-250, the bands of ∼21.4 kDa (expected size of LptE) were excised from both untreated and GI-7-treated lanes of the gel, and in-gel digestion was performed as previously described (81). LC-MS/MS of the digested samples was performed on the Thermo Scientific Orbitrap Fusion MS instrument equipped with an EASY-Spray source operated in positive ion mode (CCIC, MS&P Facility, OSU). The Thermo Scientific UltiMate 3000 RSLCnano system containing an Acclaim PepMap 100 (75 μm by 2 cm) trap column and Easy-Spray nano separation column (PepMap RSLC C_18_, 3 μM, 100 Å, 75 μm by 150 mm; Thermo Scientific) was used for injection and elution of samples. For the proteomics database search, the E. coli Uniprot protein database with trypsin/P (selected as the enzyme) was used with the fragment mass tolerance set to 0.5 Da. Mascot Daemon (Matrix Science version 2.6.0) and Scaffold Proteome software were used for the MS/MS data analysis. The results were filtered to a 1% false discovery rate and a minimum of 2 peptides per protein identification. The exponentially modified protein abundance index (emPAI) (82) was also calculated to compare the LptE levels between untreated and GI-7-treated gel fragments.

### LptD/E immunoblot analysis.

For immunoblot analysis, total membrane proteins (25 μg) resolved by SDS-PAGE under nonreducing (in the absence of β-mercaptoethanol) conditions (37) as described above were electrotransferred onto an Immun-Blot polyvinylidene difluoride (PVDF) membrane (Bio-Rad) and probed for LptD or LptE using anti-LptD (1:20,000) and anti-LptE (1:30,000) polyclonal rabbit antibodies (GenScript) and goat anti-rabbit IgG horseradish peroxidase (HRP)-conjugated secondary antibody (Sigma-Aldrich). Anti-LptD and anti-LptE antibodies were generated at GenScript using keyhole limpet hemocyanin (KLH)-conjugated peptides as the antigens (LptD epitope, QLHQKEAPGQPEPVC, and LptE epitope, ELLDKETTRKDVPSC). The epitopes were selected using the OptimumAntigen design tool (GenScript). The transferred membrane was developed with Clarity Western ECL substrate (Bio-Rad) and visualized in a FluorChem Q imager (ProteinSimple).

### LPS analysis.

To further support the results from the above-described studies showing that GI-7 affects the levels of Lpt proteins, LPS was extracted from untreated and GI-7-treated APEC (as described above) using an LPS isolation kit (BioVision, Inc., Milpitas, CA) following the manufacturer’s protocol. Extracted LPS (normalized based on the OD_600_ before extraction) was separated on a 4-to-20% gradient SDS-PAGE gel and stained with Coomassie blue R-250 to visualize the LPS levels (83). In addition, LPS was also quantitated using a total carbohydrate assay kit (Cell Biolabs, Inc., San Diego, CA) following the manufacturer’s protocol. Two independent experiments with six replicates in each experiment were performed.

### Docking studies.

Autodock 4.0 (84) was employed in GI-7 docking studies using the protein structure with PDB code 4RHB, and Discovery Studio Visualizer along with Chimera was used for protein-ligand interaction. Graphical User Interface for AutoDock Tools (ADT) was used for preparation of pdbqt files for protein and ligand and for grid box creation. AutoGrid was employed for the preparation of the grid map, and the grid size was set to 60 by 60 by 60 *xyz* points with grid spacing at 0.375 Å. Both protein and ligand were considered rigid during docking, and the outcomes of docking with 1.0 Å of positional root-mean-square deviation (RMSD) were clustered together. The docking pose with the most favorable, i.e., lowest, energy or binding affinity was aligned with the protein structure and further analyzed using Biovia Discovery Studio Visualizer. In order to determine the LptD-specific binding of GI-7, docking was also conducted with GI-2 and GI-3, which have structures similar (Fig. 1A) to that of GI-7 (18), and with GI-10 and GI-6, which have different structures (Fig. 1A) than GI-7 (18) but showed impacts against APEC infection in chickens. AutoDock Vina 1.5.6 (34) was used to further support the predicted interactions of GI-7 with LptD. PyMOL (The PyMOL Molecular Graphics System, version 2.3.4; Schrodinger LLC) was used for the visualization.

### Statistical analysis.

The statistical significance of the effects of treatments on the survival of chickens was analyzed using the log-rank test (*P* < 0.05). The statistical significance (*P* < 0.05) of the effects of treatments on the reduction of APEC lesions and APEC loads was calculated using the Mann-Whitney U test or Student’s *t* test after testing normality using the Shapiro-Wilk test. The statistical comparison of APEC lesions, APEC loads, BWGs, and FCRs between treatment groups was performed using the Kruskal-Wallis test (*P* < 0.05). The statistical comparison (*P* < 0.05) of LPS levels was performed using Student’s *t* test. GraphPad Prism 5.02 was used for the analysis.
