# Supplementary material for: Novel Small Molecule Growth Inhibitor Affecting Bacterial Outer Membrane Reduces Extraintestinal Pathogenic Escherichia coli (ExPEC) Infection in Avian Model
Source: Microbiol Spectr. 2021 Sep 1;9(2):e00006-21. doi: 10.1128/Spectrum.00006-21 (PMC8557866; doi:10.1128/Spectrum.00006-21)
Supplement: SUPPLEMENTAL FILE 1 — Supplemental material. Download SPECTRUM00006-21_Supp_1_seq12.pdf, PDF file, 1 MB. [file spectrum00006-21_supp_1_seq12.pdf]

Supplementary Materials

Cecal microbiota analysis. DNA was extracted from 0.2 g of cecal contents using PureLink^{TM} Microbiome DNA Purification Kit (Thermofisher Scientific) and treated with RNase A (Qiagen). The DNA samples were subjected to 16S rRNA V4-V5 sequencing at the molecular and cellular imaging center (MCIC) (https://mcic.osu.edu/genomics/illumina-sequencing). Amplicon libraries were prepared using IFU KAPA HiFi HotStart ReadyMixPCR Kit (Roche) and PCR clean-up was performed using Agencourt AMPure XP beads (BECKMAN COULTER Life Sciences). Nextera XT DNA Library Preparation Kit (Illumina) was used to generate Illumina library and sequencing was performed using Illumina MiSeq platform generating paired end 300-bp reads.

For the metagenomic analysis, QIIME (Quantitative Insights Into Microbial Ecology) 2 bioinformatics platform(1, 2) was used. Quality control of the raw reads was performed using FastQC 0.11.8 (Babraham Bioinformatics). Trimmomatic-0.33 was used to trim the adaptor and other illumina-specific sequences (http://www.usadellab.org/cms/?page=trimmomatic). The feature table construction and additional filtering of the sequences was performed using DADA2(2). The taxonomic analysis was performed using Naive Bayes classifiers trained on the Greengenes 13_8 99% OTUs (gg-13-8-99-515-806-nb-classifier.qza) and Silva 132 99% OTUs (silva-132-99-nb-classifier.qza) databases. The phylogenetic diversity was analyzed using align-to-tree-mafft-fasttree pipeline and alpha (Shannon's diversity index) and beta diversity (Bray-Curtis distance) were analyzed using core-metrics-phylogenetic pipeline (https://docs.qiime2.org/2019.7/tutorials/moving-pictures/).

Serum metabolome analysis. An aliquot (20 µL) of serum was mixed with 80 µL of cold methanol (MeOH) and mixture was incubated at -20°C for 30 min and precipitated proteins were

removed by ultracentrifugation at 13,000xg for 30 min. The 5 µL of supernatant was injected for each LC-MS run (2-3 runs for each sample). For quality control, equal portions of serum samples from GIs treated and control groups were pooled and run for LC-MS analysis every 8 sample runs. Metabolome profiling was performed on a Thermo Orbitrap LTQ XL in positive mode analysis with high-performance liquid chromatography (HPLC) separation on a ThermoScientific RLCS Ultimate 3000 LC system using Poroshell 120 SB-C18 (2 x 100 mm, 2.7µm particle size) column. The gradient consisted of solvent A, H_2O with 0.1% formic acid (FA), and solvent B, acetonitrile (ACN) with 0.1% formic acid at a 200 µL/min flow rate with an initial 2% B, a linear ramp to 95% B ending at 15 min, a hold of 95% solvent B for 1 min, back to 2% B within 1 min, and equilibration of 2% B until 32 min.

Data were processed using XCMS Online (https://xcmsonline.scripps.edu/). All samples were aligned with a score of 88% or above and database matching was performed using Human Metabolome Database (HMBD), selecting for adducts M+H, M+Na, M+K, and M+2H, and less than 10 ppm mass error. HMBD was used due to lack of database associated with poultry metabolites. The significantly ($P<0.05$, Kruskal-Wallis test) altered metabolites and the associated metabolic pathways were identified using XCMS Online and expressed as fold change in abundance as compared to NC (non-infected and non-treated) chickens. The principal component analysis (PCA) plot was performed to compare the metabolome profile between the treatment groups. Progenesis QI (http://www.nonlinear.com/progenesis/qi/) software was used to validate the results.

Pharmacokinetic (PK) study. The absorption and excretion kinetics of GI-7, upon oral delivery in chickens, was determined using LC-MS (CCIC, MS&P facility, OSU) as described

previously(3). Using the experimental set up as described above (floor trial), 5 chickens from each group were sacrificed at 0, 0.5, 1, 2, 4, 8, 12, 24, 84, and 180 h post treatment, blood (2 mL) was collected in EDTA tubes (BD™), and plasma was separated by centrifugation (2,000xg, 10 min) and stored at -80°C until LC-MS analysis. For the LC-MS analysis, 20 µL of plasma samples were mixed with 80 µL of cold MeOH followed by the addition of 10 µL of 1µg/mL internal drug standard (heavy labeled phenylalanine dissolved in H_2O and 0.1% FA). The mixtures were then incubated at -20°C for 30 min and proteins were removed by ultracentrifugation at 13,000xg for 25 min at 2°C. The 5 µL supernatants were run using Agilent Poroshell 120 SB-C18 column (2 x 100 mm, 2.7 µm particle size). The LC system used was a Thermo Scientific UltiMate 3000 HPLC with solvent A: 10 mM ammonium formate with 0.1 % FA and solvent B: ACN with a flow rate of 200 µL/min. Initially, the gradient was set to 2% B until 2 min and the flow was ramped after 2 min to up to 20% at 5 min, 40% at 7.5 min, and up to 90% at 9 min. The gradient was then held at 90% B until 11 min and dropped to 2% at 12 min and held there for equilibration until the run ended at 15 min. The mass spectrometer (Thermo Scientific Q Exactive Orbitrap MS) was set to parallel reaction monitoring mode (PRM) with a heated electrospray ionization source (ESI) in positive mode at 3.5 kV. The quantitation of GI-7 in plasma was performed by calibrating the standard solutions of GI-7 containing different concentrations (0.001, 0.005, 0.01, 0.05, 0.1, 0.5, 1, and 5 µg/mL) in LC-MS run as above (**Fig. S2 A, B**). Plasma samples from chickens treated with SDM were used for comparison. GI-7 was monitored at 439.2→232.1 m/z at 20 V collision energy (CE) and sulfadimethoxine at 311.1→156 m/z at 30 V CE.

Quantification of GI-7 residue in tissues: To quantify the GI-7 residue in chicken's tissues (muscle, liver, and kidney), residue analysis was conducted using LC-MS (CCIC, MS&P facility,

OSU) as described previously(4). Using the experimental set up as described above (floor trial), chickens (n=5/time point) were sacrificed at 2 days (day 13, 2DPLT), 5 days (day 16, 5DPLT), and 31 days (day 42; 31DPLT) post last treatment of GI-7. Tissues were collected in cryovials, immediately snap frozen in liquid nitrogen, and stored at -80°C until further use. The extraction solution consisting 50:50 H_2O:ACN was added to the tissues at 400 mg/mL ratio of tissue to extraction solution. Tissues were homogenized (20 on/off cycles) using metal probe sonicator device and centrifuged for 23,000 rpm for 30 min. An aliquot of 60 µL supernatant was transferred to glass vials, dried in a SpeedVac for 1.5 hours, and resuspended in 120 µL 25:25:50 H_2O:ACN:MeOH. Samples were centrifuged further for 30 min at 23,000 rpm to remove any precipitates. Samples were analyzed by LC-MS as above. The quantitation of GI-7 in chicken's tissues was performed by calibrating the standard solutions of GI-7 containing different concentrations (0.1, 0.5, 1, 5, 10, 50, 100, 500, and 1000 ng/mL) (**Fig. S2 C, D**). Tissues samples from chickens treated with SDM were used for comparison. GI-7 and SDM peaks were monitored at 439.2→232.1 m/z and 311.1→156 m/z, respectively, as above.

Table S1. Dose of growth inhibitors (GIs) administered orally in chickens.

	Weight of GI/chick/dose (µg)[†]
GI-1	173.4
GI-2	108.7
GI-3	99.6
GI-6	54.7
GI-7	109.6
GI-8	28.5
GI-9	90.7
GI-10	108.4

[†]equals to 50X *in vitro* MBC concentration in 100 µL of 25% DMSO.

Table S2. Mortality and APEC load in chickens infected with APEC using different routes and inoculation doses.

Route	Inoculum (CFU/chicken)	Mortality (%)	APEC load (average log CFU)†				
			Lung	Heart	Kidney	Liver	Spleen
S/C	10^6	0	1.4	0	1.9	0	0
	10^7	57	2.9	2.8	2.5	0.6	0
	10^8	86	2.4	0	0	0	3.1
I/T	10^6	0	3.3	0.5	3.0	0.2	1.3
	10^7	0	2.7	1.2	3.1	0.3	0
	10^8	0	3.0	0.4	0.9	0.3	1.4
I/A	10^6	14	0.8	0	1.2	0	0
	10^7	29	2.5	1.9	2.1	0.4	2.6
	10^8	14	3.4	2.0	2.5	2.0	3.4

S/C; subcutaneous, I/T: intra-tracheal, I/A; intra-airsac. [†]APEC load was quantified in three randomly selected chickens; seven chickens were included in each group.

Table S3. Primers used in this study.

Primer	Sequence (5' to 3')
LptA_F	CGGACCAGCAATCTCTTGAT
LptA_R	ACGGGTAACGACCACTTTG
LptD_F	GCGATCAGAGCGACATCTATAA
LptD_R	TGGTTAGCGGAGGCAATAC
LptC_F	TACGGACACGCTCGTCTATAA
LptC_R	GAAACGGCCTGATCGGAATAA
LptE_F	ATCCCTGCGTTTGGGTAAAG
LptE_R	TGGCGCTAATCGGGTAGATA
BamA_F	TCGGTGGTCGTCTCTTCTAT
BamA_R	CGTCACGTCTGTACCATAACTC
MlaA_F	TGATATGGCGGATGGTCTTTAC
MlaA_R	ACTGCGCACGAGTTTCTATC
LolB_F	AAGTGTACGCCCGTTTCTTC
LolB_R	GTTACCCGGTTGAGCATTCA
PbgA_F	TCAGCGTGCCGGTTATTT
PbgA_R	CGAGGCGATAAAGGCGATAA
GAPDH_F	CGGTACCGTTGAAGTGAAAGA
GAPDH_R	ACTTCGTCCCATTTCAGGTTAG

Table S4. Mean APEC load and reduction of APEC load in growth inhibitors (GIs) treated chickens.

	Mean APEC load (log reduction in APEC load†)			
Efficacy trials				
	**Liver**	**Heart**	**Lung**	**Kidney**
GI-1	6.7(-2.0)	6.8(-1.8)	6.4(-1.4)	6.6(-1.1)
GI-2	3.4(0.9)	4.1(0.8)	3.5(1.1)	3.8(1.7)
GI-3	6.5(-1.8)	7.0(-2.1)	7.0(-2.0)	6.9(-1.4)
GI-6	3.5(1.2)	3.6(1.3)	3.8(1.2)	3.7(1.9)
GI-7	2.1(2.5)*	2.7(2.3)*	2.7(2.4)*	2.3(3.2)*
GI-8	6.8(-2.2)	6.9(-2.0)	7.1(-2.0)	7.1(-1.6)
GI-9	4.7(-0.1)	5.9(-0.5)	5.6(-0.9)	5.7(-0.2)
GI-10	3.9(0.8)	3.9(1.0)	3.7(1.4)	3.1(2.4)*
PC	4.6	5.0	5.1	5.5
Dose-response trial				
GI-7 (20 mg/L)	6.0(-0.1)	6.7(-0.2)	6.3(-0.4)	5.9(0.2)
GI-7 (40 mg/L)	3.9(2.0)*	4.4(2.0)*	4.0(2.0)*	4.2(1.9)*
GI-7 (60 mg/L)	3.5(2.4)*	4.0(2.4)*	4.4(1.6)*	3.6(2.5)*
PC	5.9	6.5	6.0	6.1
Comparative efficacy trial				
GI-7	5.7(1.8)***	6.1(1.8)***	6.1(2.0)***	6.1(1.6)***
SDM	5.0(2.5)***	5.6(2.3)***	6.2(1.9)***	5.5(2.2)***
PC	7.4	7.9	8.1	7.7

†Log CFU/g tissues. PC: infected and vehicle-treated chickens, SDM: sulfadimethoxine, *$P<0.05$, ***$P<0.001$ compared to PC chickens.

Table S5. Body weight gain (BWG) of chickens infected with APEC and treated with different growth inhibitors (GIs).

	**BWG**[†]
GI-1	165.6±69.2
GI-2	125.5±34.7[b]
GI-3	254.6±95
GI-6	222.2±54
GI-7	261.3±20.4
GI-8	246.8±0.0
GI-9	214.9±31.9
GI-10	220.8±38.9
NC	279.8±13.9[a]

[†]Weight of chickens before treatment was subtracted from weight of live chickens at necropsy day.

Table S6. Microbial relative abundance (%) at family level in each individual animal of different treatment groups.

Animal Description	Relative abundance (%)											
	Microbial family											
	**A**	**B**	**C**	**D**	**E**	**F**	**G**	**H**	**I**	**J**	**K**	**L**
NC-1	0.0	0.0	0.0	0.0	0.0	76.1	0.0	4.2	0.0	19.5	0.0	0.0
NC-2	0.0	0.0	4.4	0.0	0.0	85.9	0.0	4.0	0.0	5.6	0.0	0.0
NC-3	0.0	0.0	0.0	7.1	0.0	50.6	0.0	20.6	11.2	10.2	0.0	0.0
NC-4	0.0	0.0	4.0	0.0	0.0	61.3	0.0	0.0	19.0	15.5	0.0	0.0
NC-5	0.0	0.0	4.4	0.0	0.0	73.9	0.0	5.8	12.5	3.2	0.0	0.0
GI-6-1	0.0	0.0	0.0	0.0	0.0	49.9	0.0	2.9	37.8	9.2	0.0	0.0
GI-6-2	0.0	0.0	0.0	0.0	11.7	57.4	0.0	12.9	6.9	10.8	0.0	0.0
GI-6-3	0.0	0.0	0.0	0.0	0.0	66.1	0.0	20.2	0.0	13.6	0.0	0.0
GI-6-4	0.0	0.0	3.2	5.8	0.0	60.9	4.0	8.8	5.7	11.2	0.0	0.0
GI-10-1	0.0	3.2	0.0	0.0	0.0	40.2	3.6	14.6	21.1	17.1	0.0	0.0
GI-10-2	0.0	0.0	0.0	0.0	0.0	54.6	0.0	4.4	33.5	7.3	0.0	0.0
GI-10-3	0.3	3.3	0.0	0.0	0.0	13.4	0.0	4.8	23.0	54.9	0.0	0.0
GI-10-4	0.0	0.0	0.0	4.9	1.4	46.8	0.0	11.8	8.0	26.8	0.0	0.1
GI-10-5	0.0	8.2	0.0	4.5	0.0	25.3	0.0	23.4	0.0	38.4	0.0	0.0
GI-7-1	0.0	0.0	5.4	11.5	0.0	41.6	11.1	16.9	0.0	13.2	0.0	0.0
GI-7-2	0.0	0.0	0.0	13.0	0.0	62.5	0.0	6.4	0.0	17.9	0.0	0.0
GI-7-3	0.0	3.0	8.8	7.7	0.0	49.6	5.2	16.1	3.8	5.5	0.0	0.0
GI-7-4	0.0	0.0	0.0	11.8	0.0	37.8	7.9	13.4	5.5	23.4	0.0	0.0
GI-7-5	0.0	0.0	0.0	10.9	0.0	59.6	0.0	11.5	5.3	12.5	0.0	0.0
PC-1	0.0	0.0	0.0	0.0	0.0	60.2	0.0	6.1	0.0	33.6	0.0	0.0
PC-2	0.0	0.0	0.0	0.0	0.0	51.0	0.0	5.0	28.1	15.8	0.0	0.0
PC-3	0.0	0.0	0.0	0.0	0.0	80.2	0.0	19.7	0.0	0.0	0.0	0.0
PC-4	0.0	0.0	0.0	0.0	0.0	46.7	3.6	11.9	21.3	16.3	0.0	0.0
PC-5	0.0	0.0	0.0	8.1	0.0	70.0	14.6	3.5	0.0	2.7	0.8	0.0

A, *Bifidobacteriaceae*; B, *Bacillaceae*; C, *Enterococcaceae*; D, *Lactobacillaceae*; E, *Clostridiaceae* 1; F, *Lachnospiraceae*; G, *Peptostreptococcaceae*; H, *Ruminococcaceae*; I, *Erysipelotrichaceae*; J, *Enterobacteriaceae*; K, *Non-Enterobacteriaceae Gammaproteobacteria*; L, *Unassigned*. NC: negative control (non-infected and non-treated), PC: positive control (infected and vehicle treated).

Table S7. Predicted binding energy, ligand efficiency and IC50 for LptD of different growth inhibitors (GIs) based on *in silico* docking analysis.

	**Binding Energy (kcal/mol)**	**Ligand Efficiency**	**IC_{50} for LptD (µM)**
GI-2	-6.8	0.2	11.2
GI-3	-7.3	0.3	4.8
GI-6	-4.8	0.2	330.5
GI-7	-9.1	0.3	0.2
GI-10	-5.6	0.3	77.5

References

1. Bolyen E, Rideout JR, Dillon MR, Bokulich NA, Abnet CC, Al-Ghalith GA, Alexander H, Alm EJ, Arumugam M, Asnicar F, Bai Y, Bisanz JE, Bittinger K, Brejnrod A, Brislawn CJ, Brown CT, Callahan BJ, Caraballo-Rodríguez AM, Chase J, Cope EK, Da Silva R, Diener C, Dorrestein PC, Douglas GM, Durall DM, Duvallet C, Edwardson CF, Ernst M, Estaki M, Fouquier J, Gauglitz JM, Gibbons SM, Gibson DL, Gonzalez A, Gorlick K, Guo J, Hillmann B, Holmes S, Holste H, Huttenhower C, Huttley GA, Janssen S, Jarmusch AK, Jiang L, Kaehler BD, Kang KB, Keefe CR, Keim P, Kelley ST, Knights D, et al. 2019. Reproducible, interactive, scalable and extensible microbiome data science using QIIME 2. Nature Biotechnology 37:852-857.
2. Callahan BJ, McMurdie PJ, Rosen MJ, Han AW, Johnson AJ, Holmes SP. 2016. DADA2: High-resolution sample inference from Illumina amplicon data. Nature Methods 13:581-3.
3. Sang K, Hao H, Huang L, Wang X, Yuan Z. 2016. Pharmacokinetic–Pharmacodynamic Modeling of Enrofloxacin Against Escherichia coli in Broilers. Frontiers in Veterinary Science 2:80.
4. Anna G, Andrzej P, Grzegorz T. 2014. LC-MS/MS analysis of doxycycline residues in chicken tissues after oral administration. Bulletin of the Veterinary Institute in Pulawy 58:573-579.

Supplementary figure legends

Fig. S1: A) Experimental design for efficacy assessment of growth inhibitors (GIs) in chickens. GIs were administered orally, twice a day, from day 4 to day 8. Chickens were infected (s/c; 1 × 10^7 CFU/chicken) with Rif^r APEC O78 at day 5. Clinical signs and mortality of chickens were recorded from day 5 to day 12. Chickens were euthanized and necropsied at day 12, lesions in internal organs (liver, heart, lung, and air-sacs) were scored and APEC load in internal organs (liver, heart, lung, and kidney) was quantified by plating the tissue homogenates on MacConkey agar plates containing 50 µg/mL rifampicin, **B)** Experimental design for efficacy assessment of GI-7 and sulfadimethoxine (SDM) in chickens. GI-7 (60 mg/L) and SDM (0.05%) were administered in drinking water, from day 5 to day 11. Chickens were infected (s/c; 5 x 10^6 CFU/chicken) with Rif^r APEC O78 at day 6. The amount of drinking water was adjusted daily based on the age and requirement of chickens (http://www.poultryhub.org/nutrition/nutrient-requirements/water-consumption-rates-for-chickens/). Clinical signs and mortality of chickens were recorded from day 5 to day 12. Chickens were euthanized and necropsied at day 12 and 42 and lesions and APEC load were quantified as above.

Fig. S2: Calibration of standard curves of GI-7 **(A)** and sulfadimethoxine (SDM) **(B)** for PK study. Concentrations of 0.001, 0.005, 0.01, 0.05, 0.1, 0.5, 1, and 5 µg/mL were used. Calibration of standard curves for GI-7 **(C)** and SDM **(D)** for residue analysis in tissues. Concentrations of 0.1, 0.5, 1, 5, 10, 50, 100, 500, and 1000 ng/mL were used. SDM: sulfadimethoxine.

Fig. S3: Time-kill curve of GI-7. APEC suspension adjusted to OD_{600} 0.5 was incubated (37°C, 200 rpm) with minimum bactericidal concentration (MBC) of GI-7 and viable APEC was enumerated at 0, 1, 2, 3, 4, 5, 6 and 12 h post-incubation by plating on LB agar plate.

Fig. S4: A) Shannon's diversity index measuring the microbial richness in growth inhibitors (GIs) treated, PC (infected and vehicle-treated; positive control), and NC (non-infected and non-treated; negative control) groups. $^{*}P<0.05$, Kruskal-Wallis test, **B&C)** Principal component analysis (PCA) plot comparing serum metabolome profile between GIs treated, PC, and NC groups.

Fig. S5: **A)** Body weight gain (BWG) in NC (non-infected and non-treated; negative control), PC (infected and vehicle-treated; positive control), and GI-7 and SDM treated groups. BWG was calculated by subtracting body weight of chickens at day 42 to day 1, **B)** Feed conversion ratio (FCR) in NC, PC, and GI-7 and SDM treated groups. FCR was calculated by using formula: total feed intake/BWG. SDM: sulfadimethoxine.

Fig. S6: **A)** Predicted binding of GI-7 (shown in red; top view) between β1 and β26 strands in the lateral gate of LptD, **B)** Two-dimensional picture depicting different types of potential bonding interactions of GI-7 with LptD, **C)** Three-dimensional picture depicting potential binding interactions of GI-7 with LptD.

Fig. S7: A) Predicted hydrogen bonding interactions of GI-7 with Lys234, Tyr244 and Glu733 residues in LptD as determined *in silico* by Autodock Vina, **B&C)** Predicted hydrogen bonding

interactions of GI-7 with LptD. The key amino acids of LptD predicted to be involved in hydrogen bonding interactions with GI-7 are Tyr244 and Lys234, which are next to Pro246 and Thr236, respectively at the lateral lumen gate and the channel of LptD wherein LPS enters and transported from inner membrane (IM) to outer membrane (OM) in *E. coli.*

Fig. S8: Schematic diagram showing the hypothetical mechanism of action of GI-7. After treatment, GI-7 might inhibit the transport of LPS from periplasmic space to OM by interfering the function of lipopolysaccharide transport (Lpt) complex; thus, likely depleting the LPS level at OM which is essential for bacterial growth, virulence, survival, and maintenance of permeability barrier. LPS: lipopolysaccharide; PL: phospholipids; OM: outer membrane; IM: inner membrane.

Figure S1

A

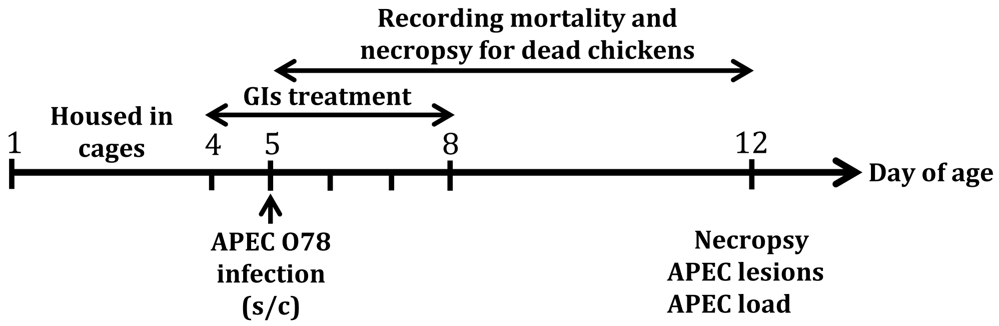
Recording mortality and
necropsy for dead chickens
GIs treatment
Housed in
cages
1
4
5
8
12
Day of age
APEC O78
infection
(s/c)
Necropsy
APEC lesions
APEC load

B

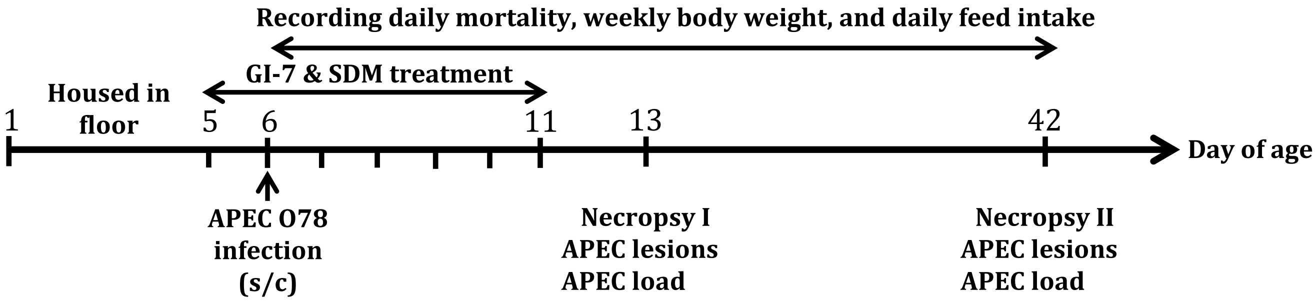
Recording daily mortality, weekly body weight, and daily feed intake
GI-7 & SDM treatment
Housed in
floor
1
5
6
11
13
42
Day of age
APEC O78
infection
(s/c)
Necropsy I
APEC lesions
APEC load
Necropsy II
APEC lesions
APEC load

Figure S2

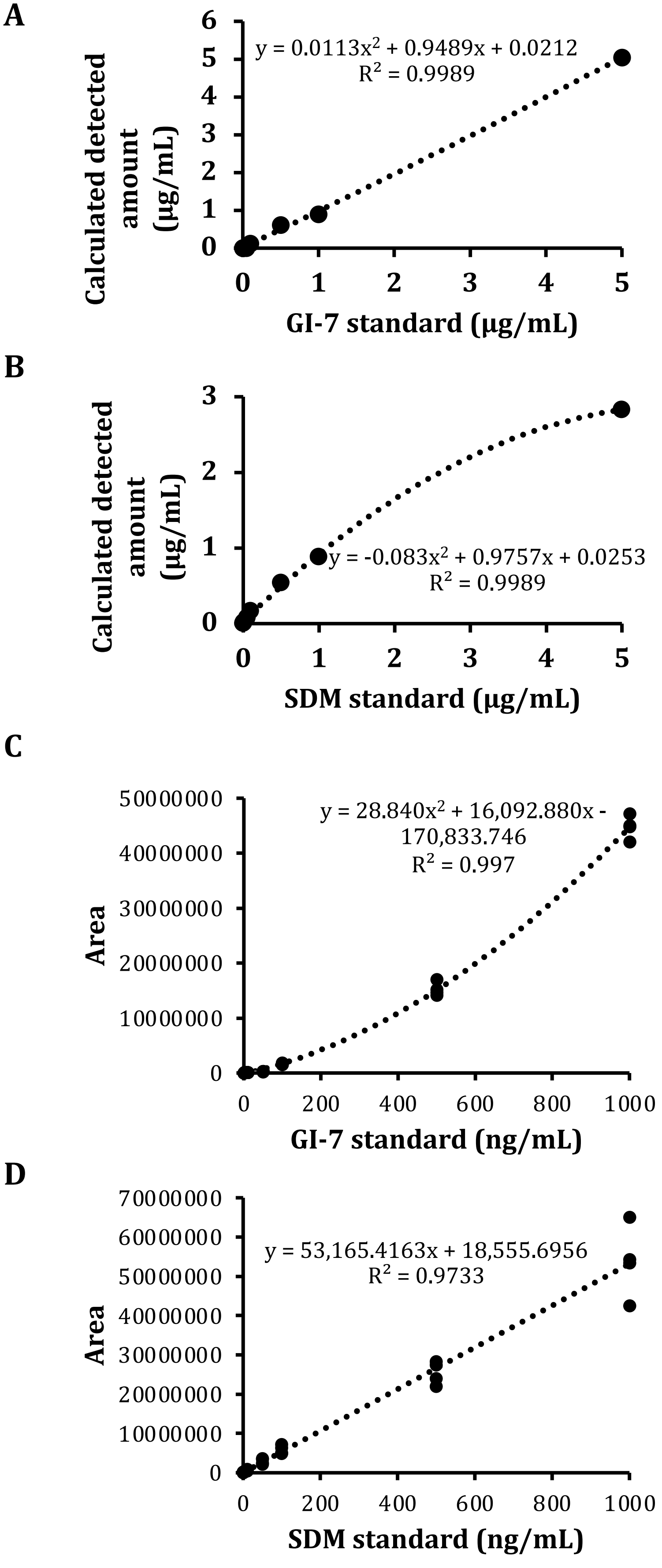
A
y = 0.0113x2 + 0.9489x + 0.0212
R2 = 0.9989
Calculated detected amount (µg/mL)
GI-7 standard (µg/mL)
B
y = -0.083x2 + 0.9757x + 0.0253
R2 = 0.9989
Calculated detected amount (µg/mL)
SDM standard (µg/mL)
C
y = 28.840x2 + 16,092.880x - 170,833.746
R2 = 0.997
Area
GI-7 standard (ng/mL)
D
y = 53,165.4163x + 18,555.6956
R2 = 0.9733
Area
SDM standard (ng/mL)

Figure S3

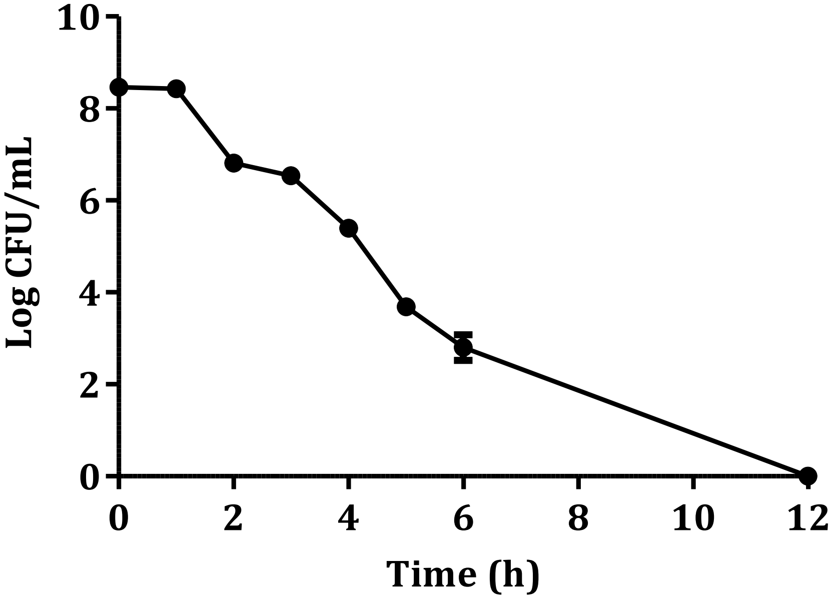

Figure S4

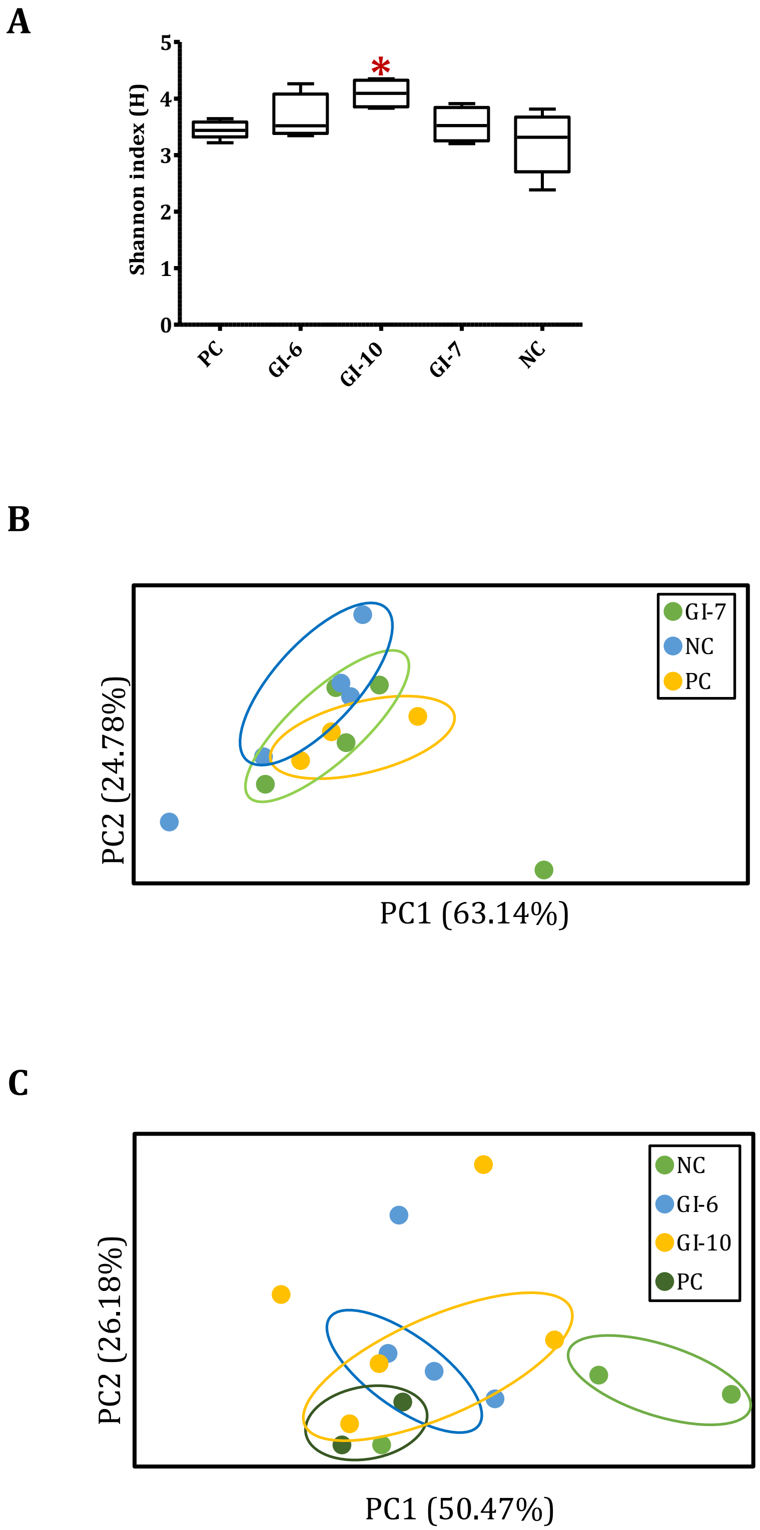
A
Shannon index (H)
0
1
2
3
4
5
PC
GI-6
GI-10
GI-7
NC
*
B
GI-7
NC
PC
PC2 (24.78%)
PC1 (63.14%)
C
NC
GI-6
GI-10
PC
PC2 (26.18%)
PC1 (50.47%)

Figure S5

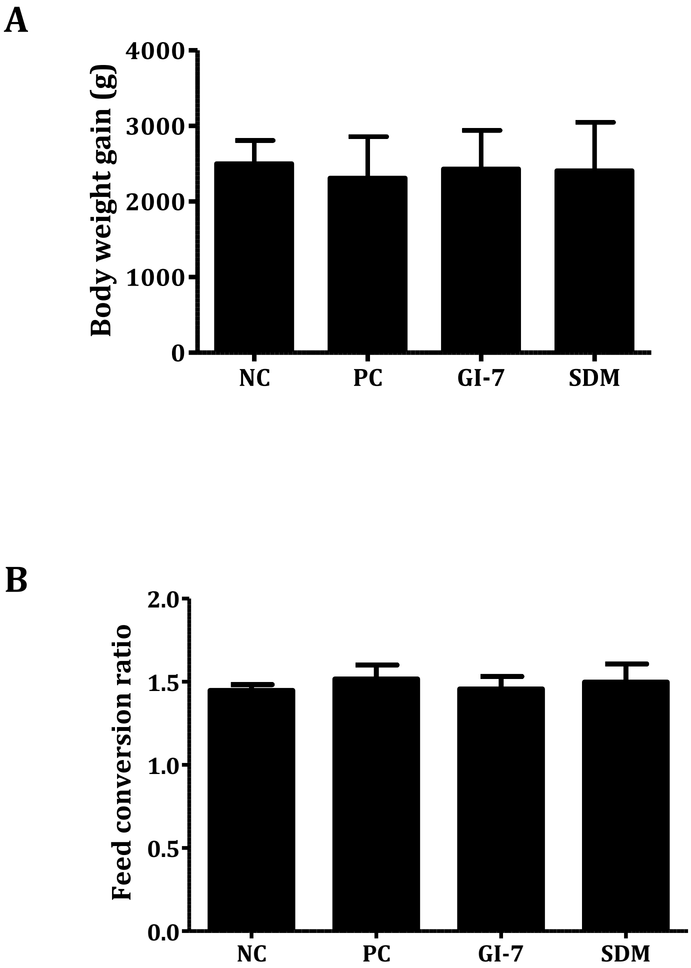
A
Body weight gain (g)
4000
3000
2000
1000
0
NC
PC
GI-7
SDM
B
Feed conversion ratio
2.0
1.5
1.0
0.5
0.0
NC
PC
GI-7
SDM

Figure S6

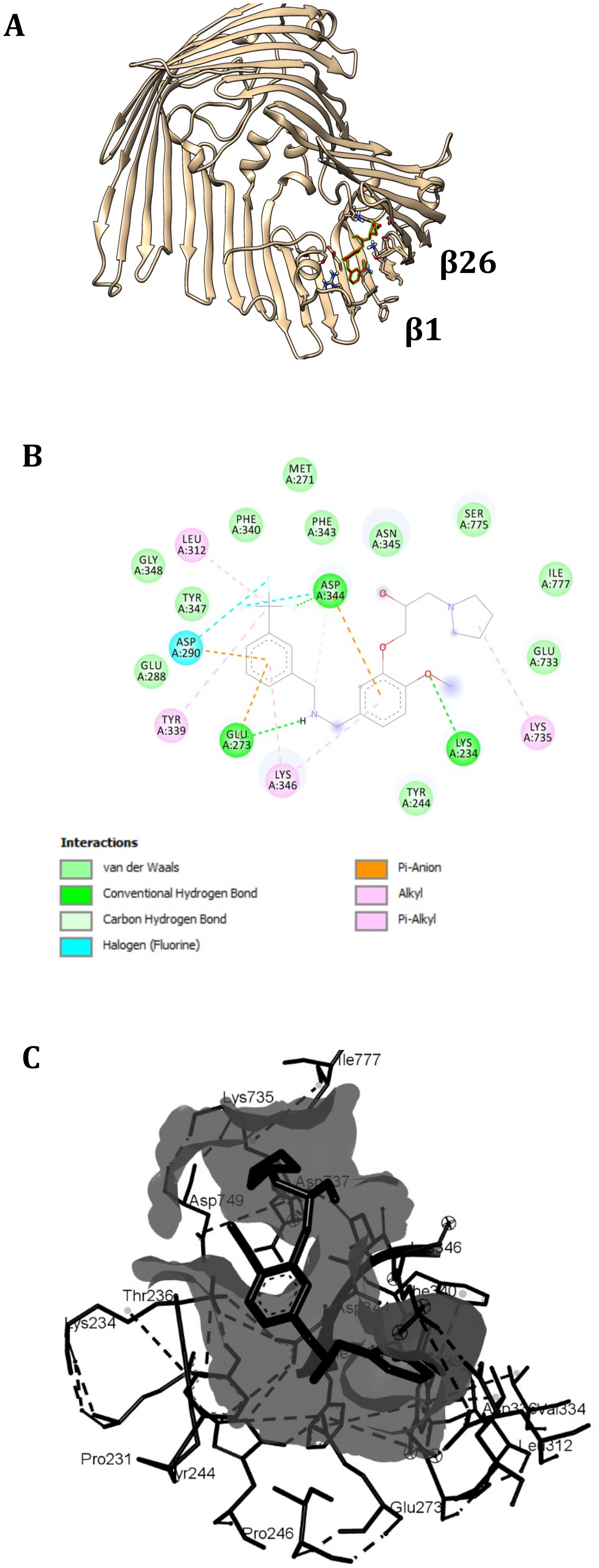
A
β26
β1
B
MET A:271
PHE A:340
PHE A:343
ASN A:345
SER A:775
LEU A:312
GLY A:348
ASP A:344
ILE A:777
TYR A:347
ASP A:290
GLU A:288
GLU A:733
TYR A:339
GLU A:273
LYS A:234
LYS A:735
LYS A:346
TYR A:244
Interactions
van der Waals
Conventional Hydrogen Bond
Carbon Hydrogen Bond
Halogen (Fluorine)
Pi-Anion
Alkyl
Pi-Alkyl
C
Ile777
Lys735
Asp737
Asp749
Thr236
Pro231
Glu273
Pro246

Figure S7

A

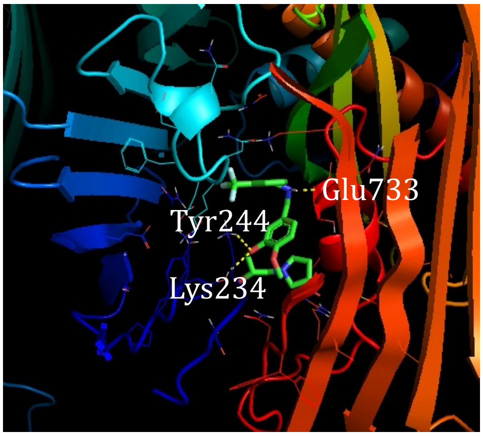

B

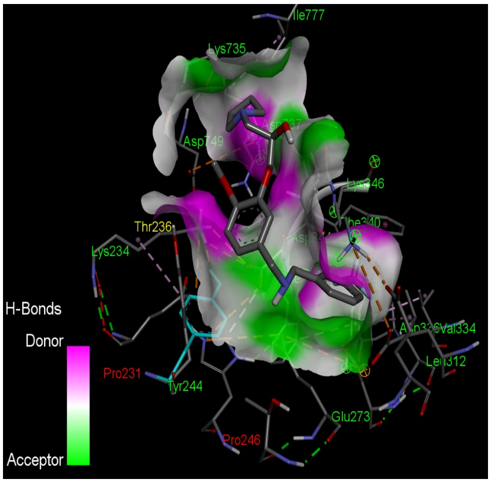

C

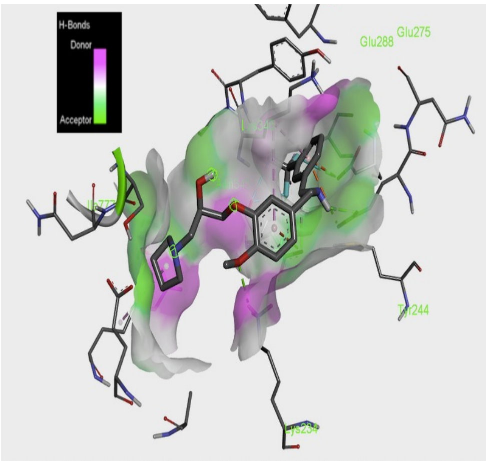

Figure S8

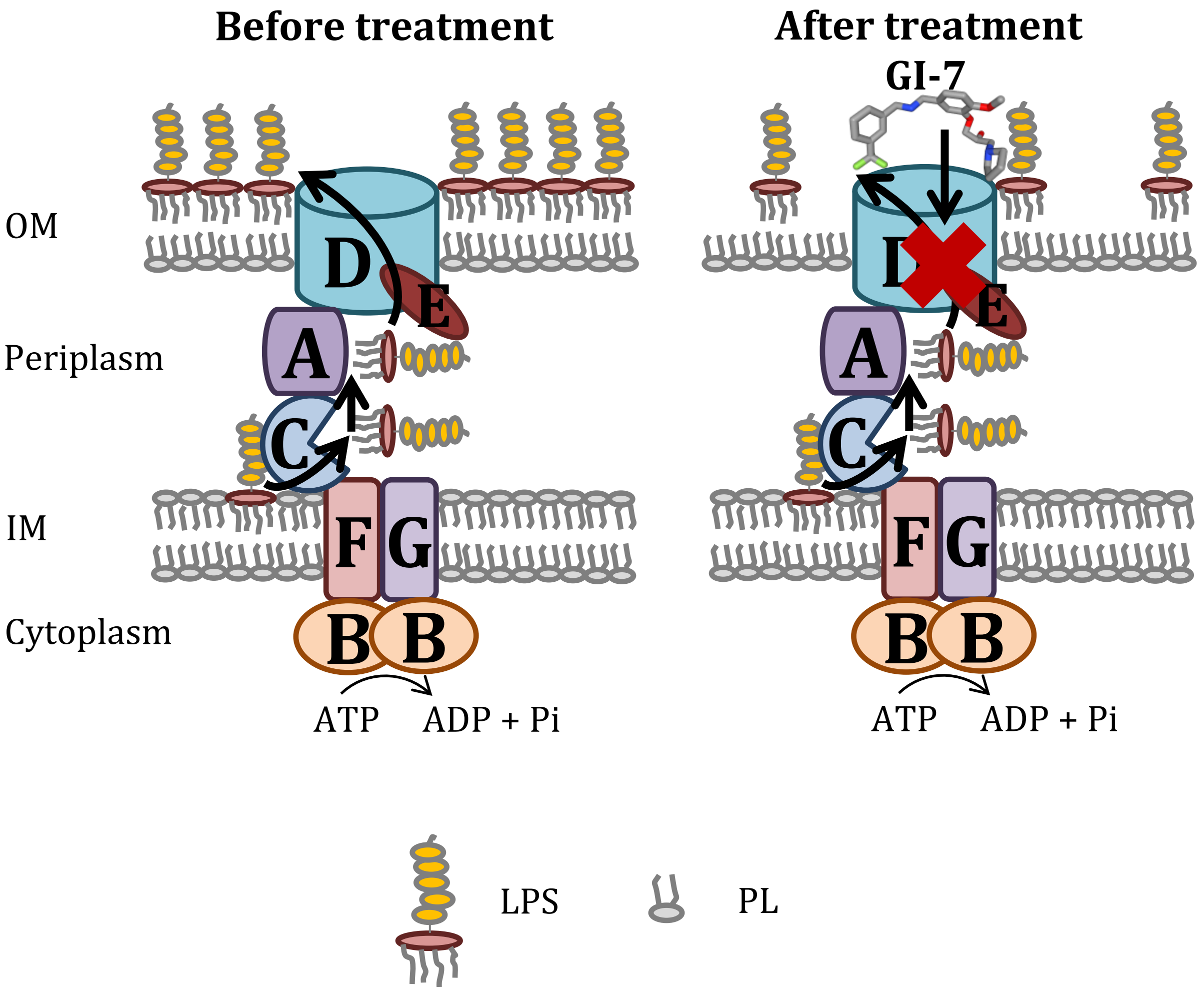
Before treatment
After treatment
GI-7
OM
Periplasm
IM
Cytoplasm
D
E
A
C
F
G
B
B
ATP
ADP + Pi
D
E
A
C
F
G
B
B
ATP
ADP + Pi
LPS
PL
